# First-in-human implementation of a bidirectional somatosensory neuroprosthetic system with wireless communication

**DOI:** 10.1186/s12984-025-01613-z

**Published:** 2025-04-23

**Authors:** Sedona R. Cady, Joris M. Lambrecht, Karina T. Dsouza, Jeremy L. Dunning, J. Robert Anderson, Kevin J. Malone, Kyle J. Chepla, Emily L. Graczyk, Dustin J. Tyler

**Affiliations:** 1 Department of Biomedical Engineering, Case Western Reserve University, Cleveland, OH 44106 USA; 2 Louis Stokes Cleveland Department of Veterans Affairs Medical Center, Cleveland, OH 44106 USA; 3 University Hospitals Cleveland Medical Center, Cleveland, OH 44106 USA; 4 MetroHealth Medical Center, Cleveland, OH 44109 USA

**Keywords:** Implanted neural interfaces, Wireless communication, Peripheral nerve stimulation, Myoelectric control, Neuroprosthesis, Upper limb loss, Intramuscular electrodes

## Abstract

**Background:**

Limitations in upper limb prosthesis function and lack of sensory feedback are major contributors to high prosthesis abandonment rates. Peripheral nerve stimulation and intramuscular recording can restore touch and relay motor intentions for individuals with upper limb loss. Percutaneous systems have enabled significant progress in implanted neural interfaces but require chronic lead maintenance and unwieldy external equipment. Fully implanted sensorimotor systems without percutaneous leads are crucial for advancing implanted neuroprosthetic technologies to long-term community use and commercialization.

**Methods:**

We present the first-in-human technical performance of the implanted Somatosensory Electrical Neurostimulation and Sensing (iSens®) system—an implanted, high-channel count myoelectric sensing and nerve stimulation system that uses wireless communication for advanced prosthetic systems. Two individuals with unilateral transradial amputations received iSens® with four 16-channel composite Flat Interface Nerve Electrodes (C-FINEs) and four Tetra Intramuscular (TIM) electrodes. This study achieved two key objectives to demonstrate system feasibility prior to long-term community use: (1) evaluating the chronic stability of extraneural cuff electrodes, intramuscular electrodes, and active implantable devices in a wirelessly connected system and (2) assessing the impacts of peripheral nerve stimulation on three degree-of-freedom controller performance in a wirelessly connected system to validate iSens® as a bidirectional interface.

**Results:**

Similar to prior percutaneous systems, we demonstrate chronically stable extraneural cuff electrodes and intramuscular electrodes in a wirelessly connected implanted system for more than two years in one participant and four months in the second participant, whose iSens® system was explanted due to an infection of unknown origin. Using an artificial neural network controller trained on implanted electromyographic data collected during known hand movements, one participant commanded a virtual hand and sensorized prosthesis in 3 degrees-of-freedom. The iSens® system simultaneously produced stimulation for sensation while recording high resolution muscle activity for real-time control. Although restored sensation did not significantly improve initial trials of prosthetic controller performance, the participant reported that sensation was helpful for functional tasks.

**Conclusions:**

This case series describes a wirelessly connected, bidirectional neuroprosthetic system with somatosensory feedback and advanced myoelectric prosthetic control that is ready for implementation in long-term home use clinical trials.

*Trial registration:* ClinicalTrials.gov ID: NCT04430218, 2020-06-30.

**Supplementary Information:**

The online version contains supplementary material available at 10.1186/s12984-025-01613-z.

## Background

Loss of a hand due to amputation results in impaired dexterity [1], higher rates of mental health disorders [2], and social insecurities that impact relationships [3]. Use of body-powered and myoelectric prostheses can reduce disability in individuals with upper limb loss [4]. Despite advances in prosthesis mechatronics and control algorithms that aim to improve device function and acceptance, prosthesis abandonment still occurs at high rates. Prior studies have shown that limited functionality, lack of sensory feedback, heavy device weight, and user discomfort are major contributors to prosthesis abandonment [5–9]. Addressing unmet prosthesis user interface needs, i.e. control and sensory feedback, is required to challenge prosthesis abandonment.

Traditional upper limb myoelectric prosthetic controllers use two surface electromyogram (EMG) inputs from agonist and antagonist muscles to move the prosthesis unidirectionally in 1 degree-of-freedom (DOF) [10], typically opening or closing the hand. Several commercially available prostheses allow for sequential movement in more than 1 DOF by using a unique movement pattern, such as co-contraction or two quick identical motions, to switch between movement along different joint angles or to update the prosthetic grip position [11]. To enable more intuitive movement in multiple DOFs, commercially available “pattern recognition” machine learning algorithms map surface EMG inputs from multiple electrode sites to prosthesis hand postures or DOFs [11]. However, surface EMG recordings vary based on arm posture and electrode position [12], resulting in unreliable and reduced controller performance [13]. Poor selectivity of surface EMG electrodes results in increased channel crosstalk that can reduce myoelectric decoding accuracy [14], particularly affecting the ability to decode unique DOFs. Pattern recognition algorithms with surface EMG inputs often require users to calibrate controllers daily or multiple times per day to account for variations in electrode position, fluctuations in weight, or skin sweat.

Percutaneous interfaces with implanted EMG electrodes demonstrate improved stability, selectivity, and controller performance when compared to surface EMG [14, 15]. Additionally, implanted EMG electrodes provide stable myoelectric controller inputs without needing to collect additional EMG training data for months [14, 16–18]. Implanted myoelectric sensing using percutaneously connected Tetra Intramuscular electrodes (TIMs) has provided stable, high fidelity myoelectric recording that supports 3–4 DOF controller inputs for up to 7 years in human subjects [14, 16]. TIMs have supported high DOF myoelectric controllers tested in virtual environments, but they have not been functionally tested with real-time upper limb prostheses.

In addition to improved control and functionality, upper limb prosthesis users desire sensory feedback in an ideal prosthetic device [6, 19, 20]. Somatosensation provides feedback about our environment, contributing to emotional connection, social interaction [21], and error correction during dexterous movements [22]. Without touch feedback, upper limb prosthesis users rely significantly on vision when manipulating objects compared to able-bodied individuals [23].

A limited number of commercial prosthetic devices, such as the DEKA LUKE arm (Mobius Bionics, Manchester, NH, USA) [24], the Ability Hand (PSYONIC, San Diego, CA, USA) [25], and the VINCENTevolution4 (Vincent Systems, Karlsruhe, Germany) [26], incorporate sensory feedback by applying vibrotactile sensation to skin on the proximal residual limb. Users learn to associate haptic feedback on the proximal arm with sensors located on the hand region of the prosthesis, but the mismatched location and quality may increase cognitive load [27]. Eliciting intuitive touch information instead requires activating somatosensory pathways that map directly to the missing hand region.

Directly activating peripheral nerves with transcutaneous or implanted electrodes can create touch perceptions in the missing hand region, matching visual feedback of objects interacting with prosthetic hands to restore several characteristics of sensory feedback [28–30]. Implantable peripheral nerve stimulation devices offer stable interfaces that eliminate the burden of daily placement of external electrodes [17, 31, 32]. Peripheral nerve stimulation via extraneural and intraneural electrodes allows for the activation of sensory axons that evoke touch perceptions of varying intensities, qualities, and locations in the upper extremity [17, 28, 29, 31, 33]. Touch feedback via implanted nerve electrodes reduces phantom limb pain [17, 32], improves grip force modulation [28, 29] and object discrimination [29, 34], and positively impacts emotional and psychosocial outcomes [35–39]. Extraneural cuff electrodes such as Flat Interface Nerve Electrodes (FINEs) have been implanted in individuals with upper limb loss for up to 12 years, demonstrating chronic stability required for long-term use of sensory restoration systems [28, 31].

Previous studies investigating implanted nerve and myoelectric recording electrodes for upper limb loss involved percutaneous systems. Percutaneous leads exiting through skin on the arm or osseointegrated conductors connect implanted electrodes to an external neural interface processor, providing wired connections for communication and power. Percutaneous leads present drawbacks such as lead maintenance, high wire count crossing the skin, infection risk, and the burden of carrying an external neural interface processor, factors that may deter long-term adoption of implanted bidirectional neural interfaces. Although the risk of infection for coiled percutaneous leads is low [40], the area must be carefully maintained and protected to prevent infection, skin irritation, and lead pulling. Furthermore, the surface area available for each percutaneous exit site limits the number of stimulating and recording electrodes. Osseointegrated interfaces allow for a more compact external system placed between the abutment and prosthesis [41] but also require careful maintenance to prevent infections and irritation [42].

In two short-term home trials, individuals with implanted percutaneous FINEs used an external stimulator and single degree-of-freedom (DOF) surface EMG prosthesis with force sensors [35–37]. Use of the sensory-enabled myoelectric prosthesis at home yielded significant improvements in psychosocial outcomes such as self-efficacy, embodiment, and quality of life. However, the implanted percutaneous system used in this study is not ideal for long-term commercial use due to the challenges associated with percutaneous leads. Fully implantable devices that eliminate percutaneous leads are necessary to move towards translation of implanted neural interfaces and bidirectional upper limb prostheses for long-term community use.

**Fig. 1:**
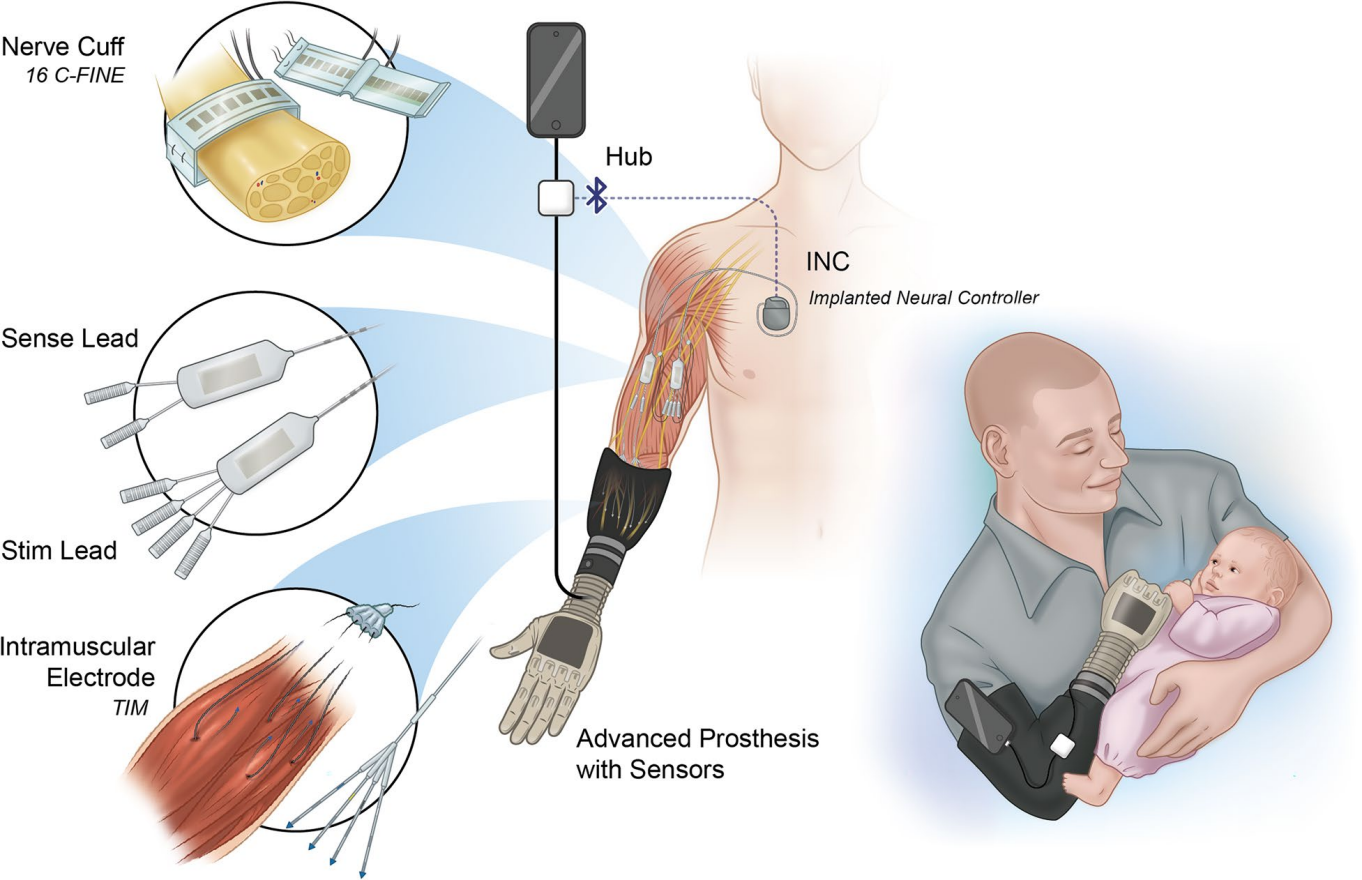
iSens® system implanted and external components. (Left) Overview of the iSens® system implanted components. The system can support up to four Smart Leads, but only two are shown. In the example illustration, the INC powers and enables communication to one Smart Stim Lead and one Smart Sense Lead via a bifurcated lead. The Smart Stim Lead connects to two 16 C-FINEs, and the Smart Sense Lead connects to two TIMs. (Right) External system components consist of a BLE-connected Hub with wired connections to a phone and advanced prosthesis with sensors

**Fig. 2:**
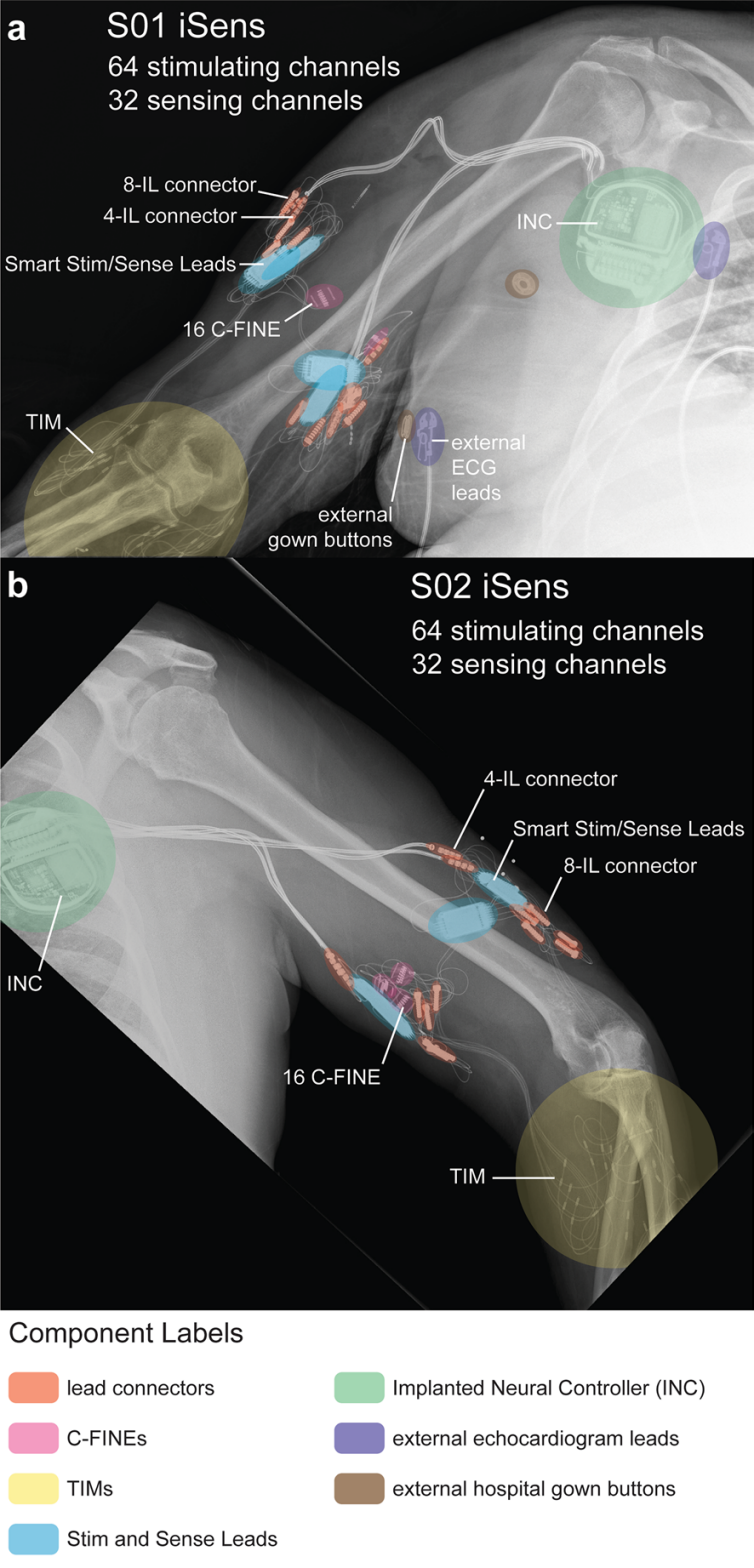
iSens® X-rays. X-rays showing iSens® implanted components in **a** S01 and **b** S02. To improve lead organization compared to S01’s original surgery, implanted device components and connectors in S02’s system were deliberately routed along the same direction, and excess lead length was intentionally organized to reduce bulk

With this motivation, our team developed a peripheral nerve stimulation and myoelectric sensing device with wireless communication: the implanted Somatosensory Electrical Neurostimulation and Sensing (iSens®) system (Figs. 1, 2) [43]. The iSens® system integrates stimulating and recording electrodes from previous work with active implantable devices to eliminate the percutaneous interface and allow for an increased number of implanted channels.

Therefore, this study addressed two essential aims to drive translation of fully implantable devices for upper limb loss. First, we evaluated the chronic stability of iSens® extraneural cuff electrodes, intramuscular electrodes, and active implantable devices to validate long-term function of a wirelessly connected sensorimotor implanted system. We hypothesized that C-FINE contacts would demonstrate stable tissue resistance and stimulation thresholds and that TIM electrode arrays would demonstrate stable channel crosstalk. Second, we determined the effects of peripheral nerve stimulation on 3 DOF controller performance to validate the simultaneous utilization of C-FINEs and TIMs in a wirelessly connected, bidirectional neuroprosthetic device. We hypothesized that applying stimulated sensory feedback would result in improved or equivalent functional performance while using a real-time, 3 DOF myoelectric prosthetic controller for tasks of daily living compared to no stimulation.

Non-percutaneous, fully implantable neural interfaces are required for translating implanted devices to long-term home use. This is the first work showing simultaneous use of peripheral nerve stimulation and myoelectric sensing in a wirelessly connected interface. In this study, we present a necessary validation step prior to testing iSens® in long-term clinical trials: the technical feasibility of iSens® in human subjects to validate utilizing a wirelessly connected implanted sensorimotor system for restoring somatosensation and functional prosthetic control.

## Methods

### Study design

Inclusion criteria consisted of medically stable individuals with upper limb loss, fully healed residual limb surgical sites, and viable peripheral nerve function. Exclusion criteria consisted of the following: counterindications preventing surgery, women who were pregnant or unwilling to prevent pregnancy during the study, uncontrolled diabetes, active infections, sores on the amputated limb, or the inability to provide informed consent.

Two participants with unilateral transradial amputations enrolled in the study. S01 is a 55-year-old male with a right-handed transradial amputation from a traumatic accident in 2004. S01’s residual limb extends 15 cm distal to the elbow. Prior to enrolling in this study, S01 received a percutaneous peripheral nerve stimulation implant in 2013, which he maintained for 8 years.

S02 is a 63-year-old male with a left-handed transradial amputation from a traumatic accident in 2018. S02’s residual limb extends 24 cm distal to the elbow, almost spanning to the wrist. S02 had no prior experience with peripheral nerve stimulation.

Both participants had no prior experience with implanted myoelectric control. S01 was an active user of myoelectric prostheses with two-site agonist–antagonist control. S02 primarily used a body-powered prosthetic device, but he had experience with two-site agonist–antagonist myoelectric prostheses. The study procedures were approved under a Food and Drug Administration Investigational Device Exemption, the Department of Veterans Affairs Medical Center Central Institutional Review Board, and the Department of the Navy Human Research Protection Program. Subjects provided written informed consent for the study.

### iSens® system description

The iSens® system [43] is a non-percutaneous, inductively recharged, peripheral nerve stimulation and intramuscular recording device with wireless communication, intended to support 64 stimulating and 16 bipolar myoelectric sensing channels (Fig. 1). A wireless Bluetooth Low Energy (BLE) connection communicates between an implanted neural controller (INC) and an external Hub that connects to a portable user interface and prosthesis. Adapted from the Medtronic Intellis™, the INC communicates wirelessly with the Hub and powers four Smart Leads, including two Smart Stim and two Smart Sense modules [43]. Each Smart Stim module controls 32 channels of stimulation by connecting to two 16-channel C-FINEs [44] via four 8-channel inline connectors. Each Smart Sense module consists of 16 channels configured into eight bipolar pairs and connects to two TIMs [45] through two 8-channel inline connectors.

### Surgical implantation

S01, a pioneer in the percutaneous sensory system [28, 31], had the original system completely removed and replaced with the iSens® system during a single operation. S01’s percutaneous system consisted of three 8-channel FINEs located proximal to the elbow and percutaneous leads exiting through the lateral upper arm. The three-hour explant surgery included one incision near the percutaneous exit site matrix in the upper arm. Percutaneous leads were removed by cutting the leads inside of the body and then pulling each lead from the outside to prevent infection. Spring-and-pin connectors [46] that linked percutaneous leads to FINEs were bundled into an encapsulated mass near the percutaneous exit sites. The connector mass was removed by cutting leads and dissecting around the mass. FINEs were removed by dissecting around encapsulation.

Surgeons implanted S01 with the iSens® system within five hours, starting distally and progressing proximally (Fig. 2a). The iSens® surgery included a total of five incisions: two incisions distal to the elbow, two incisions in the upper arm (anteromedial and posterolateral), and one incision in the chest. Two incisions distal to the elbow facilitated implanting four TIMs, totaling 16 bipolar recording channels (Supplementary Table S1, Additional File 1). Targeted muscles were pre-identified via palpation prior to surgery. TIMs were inserted into each target muscle with an insertion tool, barbed end angled proximally, and tunneled to the posterolateral and anteromedial incisions proximal to the elbow. Four 16-channel C-FINEs were implanted, totaling 64 stimulating channels (Supplementary Table S1, Additional File 1). Placements of the distal median nerve and radial nerve C-FINEs matched the locations of the explanted FINEs. The proximal median nerve C-FINE lead connected to the same Smart Stim as the radial nerve C-FINE by tunneling the lead to the posterolateral incision. An incision was made in the right chest, and two Medtronic 8-2-4 bifurcated extension leads were tunneled from the posterolateral and anteromedial incisions in the arm towards the chest incision. Smart Sense and Stim Leads were introduced to connect to TIMs and C-FINEs, respectively, as well as to bifurcated leads to connect the INC. The INC was sutured under the skin near the pectoralis major muscle. The BLE connection between the Hub and INC was sufficient to traverse the sterile field. Using custom MATLAB-based software (The MathWorks® Inc., Natick, MA, USA) on a laptop connected to the Hub, we performed tissue resistance measurements and recorded EMG to verify the INC’s connection to each Smart Lead.

Twenty-two weeks after the original surgery, two of S01’s Smart Leads stopped communicating with the INC due to a suspected failure in the anteromedial bifurcated lead. A four-hour revision surgery occurred 62 weeks after S01’s original surgery, which included replacing the suspect bifurcated lead, moving the INC more medially, and rerouting bifurcated leads away from the axilla. Three incisions were made in the right chest near the INC, the anteromedial upper arm near the Smart Leads, and a superolateral location in the upper arm for bifurcated lead rerouting. Replacing the suspect bifurcated lead restored communication with the Smart Sense, but the Smart Stim remained unresponsive. Due to surgical risks, the Smart Stim was left unpowered in the body, and the study proceeded with S01’s system consisting of two Smart Sense Leads, one Smart Stim Lead, four TIMs, the proximal median nerve C-FINE, and the radial nerve C-FINE.

S02’s surgery duration was 5.25 hours to implant four C-FINEs, four TIMs, four Smart Leads, and one INC (Supplementary Table S1, Additional File 1). Implanted components were deliberately routed via nine incisions for improved lead organization (Fig. 2b). Two incision strategies differed in S02’s surgery compared to S01’s original implant. First, surgeons made five incisions distal to the elbow for TIM implants: three near extensor muscles and two near flexor muscles. Also, an incision near the anterior shoulder enabled routing bifurcated leads further away from the axilla. Surgeons explanted S02 15 weeks post-implant due to an infection of unknown origin.

### Data reporting time intervals

This study reports on the initial results with S01 over the first 27 months after his implant and S02’s 15 weeks of participation prior to his explant. Study participants completed experimental sessions approximately monthly, but the data collection schedule was often governed by the participants’ personal availabilities, their health, and the amount of time required to run each experiment. For S01, we are reporting on the first 26 months for stimulation threshold charge and percept stability analyses and 27 months for tissue resistance stability, EMG stability, and active implantable device stability analyses. S01’s EMG stimulation artifact testing occurred 20-months post-implant, virtual controller testing occurred 21-months post-implant, and functional controller testing occurred across two sessions at 20- and 21-months post-implant. For S02, we are reporting on the first 10 weeks for EMG stability and 15 weeks for stimulation threshold charge stability, tissue resistance stability, percept stability, and active implantable device stability. S02’s EMG stimulation artifact testing occurred at 10-weeks post-implant.

### Experimental setup

Peripheral nerve stimulation and myoelectric control experiments commenced two- and four-weeks post-implant, respectively, to allow the electrode-tissue interface to heal. Peripheral nerve stimulation and EMG recording were performed via custom MATLAB graphical user interfaces that controlled the Hub. Participants interacted with a Wacom® tablet PC monitor for controller training and reporting sensory percepts (Cintiq Pro 24, Wacom International, USA).

### iSens® communication validation

Hub-INC wireless communication and INC-Smart Lead wired communication were each assigned a binary status of successful or unsuccessful at each experimental encounter. Reception of BLE Received Signal Strength Indicator (RSSI) and battery level in the Hub user interface confirmed Hub-INC communication. INC-Smart Lead wired communication was assessed via the Hub user interface by confirming successful discovery of each Smart Lead by the INC.

### Peripheral nerve stimulation

Somatosensory percepts produced by peripheral nerve stimulation were described in terms of location, quality, and intensity for each of 15 individual contacts in each C-FINE. Stimulation consisted of biphasic, cathode-first, charge-balanced rectangular pulse trains with an anodic phase twice the width of the cathodic phase. Percepts reported in this study resulted from stimulation between one cathode and one anode at perceptual threshold, or the minimum charge required for sensory detection. To measure perceptual thresholds and tissue resistance, one of the fifteen 0.5 mm^2^ electrode contacts was set as the cathode, and the anode was set as the two electrically connected 2 mm^2^ electrode strips, or “anode strip.” We measured perceptual threshold charge by first stimulating at 100 Hz and 250 μs while increasing pulse amplitude in 0.01 mA steps until the participant reported sensation. Next, we maintained this pulse amplitude and decreased pulse width, performing a binary search to find the minimum pulse width that evoked a sensory percept within 10 μs [31]. Due to time constraints, we completed one trial per contact during each session. Charge safety limit was set to 250 nC based on the C-FINE’s electrode surface area [47] and a Shannon curve limit of k = 1.1 [48].

Tissue resistance values were measured by passing a sub-threshold rectangular pulse waveform at 1000 Hz, 70 μs, and 0.2 mA between each contact and corresponding anode, averaging the result across eight trials per contact during each session. Five months after S01 received iSens®, tissue resistance measurement procedures were modified to stimulate at lower pulse amplitude levels to avoid motor activation and pain thresholds. Sufficient tissue resistance data was available to analyze trends across time for two C-FINEs in S01 and two C-FINEs in S02. C-FINE chronic stability was assessed by comparing linear regression slopes of perceptual threshold charge and tissue resistance over time to constant models with F-tests. C-FINE contacts with any resistance measurement exceeding 15 kΩ, likely indicating a loose connection between the C-FINE contact and Smart Stim, were excluded from analyses.

Participants drew on upper limb template images to describe perceived locations and verbally reported as many self-chosen quality descriptors as needed to define sensation quality. The percent of contacts per C-FINE evoking percepts on the hand was assessed across time by fitting logistic regression models and comparing to constant models with Chi-squared tests. For location analyses, we excluded percepts in which only proprioceptive qualities were reported. The percent of contacts evoking hand percepts across all time points were compared between C-FINEs with one-way Kruskal–Wallis tests and Tukey’s multiple comparison adjustment. Location stability for each C-FINE contact was assessed by the mean Jaccard similarity coefficient for each combination of location drawings across all experimental sessions. Jaccard similarity, defined as the intersection of selected pixels divided by the union of selected pixels, measures image overlap and therefore can estimate location stability. Differences in location stability were assessed between S01’s four C-FINEs with a Kruskal–Wallis test and Tukey’s multiple comparison adjustment, and stability differences between S02’s two C-FINEs were assessed with a Wilcoxon rank-sum test.

We computed the percent of C-FINE contacts in which participants reported each quality descriptor across time. Since participants could report multiple qualities per contact, quality percents could sum to more than 100%. Stability per quality descriptor was evaluated by fitting the percent of contacts associated with each descriptor across time to logistic regression models and comparing to constant models with Chi-squared tests. Next, we categorized quality terms as tactile, proprioceptive, or pain descriptors. The percent of contacts in each descriptor category was measured across time by fitting logistic regression models and performing Chi-squared tests. The percent of contacts in each descriptor category were grouped across all time points and compared with one-way Kruskal–Wallis tests with Tukey’s multiple comparison adjustments.

### Raw EMG analysis

iSens® Smart Sense modules can record raw EMG at maximum resolutions of 8-bits for eight channels at a time or 16-bits for four channels at a time. The input-referred resolution depends on the set gain and ranges between 10–100 μV for 8-bit resolution and 0.04–0.4 μV for 16-bit resolution. We evaluated iSens® myoelectric recording crosstalk across experimental sessions by recording raw EMG signals at 1 kHz and 8-bit resolution in batches of eight EMG channels per Smart Sense to compare crosstalk across the maximum number of Smart Sense channels. While recording, participants moved to 18 hand postures at self-chosen paces. The system applied a 15–350 Hz bandpass filter, and filtered EMG signals were transmitted via BLE from the INC to the Hub. Raw EMG features, including waveform length (WFL) and mean absolute value (MAV), were post-hoc calculated with 100 ms windows to classify EMG channels as responsive or unresponsive/noise-only recordings. WFL is the sum of the absolute value of change in the EMG signal per time point within the window divided by the window length [49], and MAV is a sliding average of the absolute value of the EMG signal. To identify unresponsive EMG channels, we categorized channels with low WFL variance (threshold: 0.02 μV), three or less WFL peaks across the signal exceeding three times the baseline noise level, and at least 90% of MAV below 17 μV as noise-only recordings. EMG channel crosstalk was estimated by calculating the cross correlation between channels across the entirety of the signal. Chronic stability of Smart Sense crosstalk was evaluated by comparing linear regression slopes of mean channel cross correlation across time to constant models.

To evaluate the impact of stimulation on myoelectric sensing, we recorded raw EMG signals at 1 kHz in batches of four channels to obtain the highest signal amplitude resolution of 16-bits for spectral analyses. Participants executed 18 postures at their own paces while raw EMG was recorded, with and without stimulation using median nerve C-FINE contacts eliciting tactile percepts on the hand. Three stimulation contacts were tested for S01, and two contacts were tested for S02. Stimulation parameters were set at 70 Hz, 250 μs, and variable pulse amplitude for a mid-comfortable intensity. The frequency of 70 Hz was chosen as it is within the normal frequency range used for real-time stimulated sensory feedback and it avoids both the United States power line frequency (60 Hz) and the Smart Sense wired packet rate (100 Hz), allowing for clear identification of the stimulation frequency and harmonic frequencies when performing EMG frequency spectral analysis. The pulse width 250 μs was used to match the parameters used during real-time controller testing. WFL was post-hoc calculated from raw EMG using a 100 ms window to show the impact of stimulation on EMG features used for controller algorithms.

EMG channels were classified into the following categories: (1) not affected by stimulation, (2) increased overall signal power during stimulation, (3) intermittent stimulation artifact, (4) consistent stimulation artifact, or (5) unresponsive.

Power spectra of frequencies across time, represented by spectrograms, were obtained from each time domain raw EMG signal by applying short-time Fourier transforms with a 500 ms window. For each EMG channel recording with stimulation, we found the median power at the 70 Hz stimulation frequency across all time points and subtracted the median power at neighboring frequencies across all time points, within the ranges 60–65 Hz and 75–80 Hz. We repeated the same process for each EMG channel recording without stimulation. Next, we found the ratio of 70 Hz power increased with stimulation compared to without stimulation to obtain the ratio-of-frequency-power-increased, ϕ_f | f=70 Hz_. Additionally, we found the ratio-of-frequency-power-increased, ϕ_f_ , at all 70 Hz harmonics: 140 Hz, 210 Hz, 280 Hz, 350 Hz, 420 Hz, and 490 Hz.

Next, we measured the overall EMG frequency power change when stimulation was present. EMG frequency power has been shown to relate to different contraction forces [50–52], so an increase in overall EMG signal power may indicate voluntary changes in muscle tension. Across all time points and frequencies other than 70 Hz and harmonics, we calculated the ratio of median signal power with stimulation compared to median signal power without stimulation to obtain the ratio-of-overall-power-increased, ϕ_overall_. Within the range of 10–500 Hz, we excluded frequencies within ± 5 Hz of the 70 Hz stimulation frequency and harmonics.

EMG channels demonstrating low ϕ_f_ (threshold: 15), low ϕ_overall_ (threshold: 15), and high WFL variance during attempted movements with and without stimulation were categorized as unaffected by stimulation.

### Prosthetic controller development and testing

*Controller training and development:* Both participants collected EMG training data for myoelectric controller development. However, only S01 tested myoelectric controllers in real-time, as S02’s iSens® system was explanted before testing commenced.

The iSens® system streams 16 channels of EMG features with 10-bit resolution, calculated from 1 kHz and 32-bit resolution raw data across 100 ms bins at a 50 ms update rate. Data collected by the INC from both Smart Sense Leads were transmitted to the Hub and streamed to a PC. EMG channels that demonstrated high stimulation artifact, discussed further in the Results section, were excluded from controller training and development.

A real-time MATLAB-Simulink interface automated the collection EMG feature data for controller training. WFL features were recorded while S01 performed 10 repetitions of 18 hand postures, for a total of 180 trials. A screen displayed a virtual hand to prompt each target posture [14, 16], which included all single and paired combinations of the following DOFs: hand open-close, wrist flex-extend, and pronate-supinate. This study utilized WFL as the controller input [49] since it is robust to changes in baseline raw EMG, whereas MAV recorded by iSens® demonstrated non-zero baseline values that could be accounted for through calibration but did not remain at consistent levels across sessions. The target posture was represented as a 3-element direction vector, in which each element corresponded to a positive (+ 1), neutral (0), or negative (− 1) DOF direction. Next, the 3-element direction vector was scaled by mean WFL amplitude across all 16 EMG channels at each time point to estimate user effort, corresponding to a 3-element effort vector.

We developed an artificial neural network (ANN) controller using WFL from EMG channels as training set inputs and the 3-element effort vector as training set outputs at each time point per trial repetition. Of the ten movement repetitions for each target posture, three repetitions least similar in EMG activation patterns were excluded from training, four repetitions comprised the training dataset, and three repetitions comprised the validation dataset. Using MATLAB’s Machine Learning Toolbox, we developed a simultaneous and proportional 3 DOF ANN controller with 14 hidden nodes for S01. During real-time use, the ANN mapped EMG WFL features to continuous intended joint angle velocities in each DOF simultaneously. The joint angle velocity for each DOF direction was independently adjusted by a configurable gain and threshold to the participant’s preference.

*Virtual environment performance:* We evaluated S01’s controller performance with and without stimulation to validate EMG channel stimulation artifact rejection and to determine if relaying tactile feedback, in place of proprioception, would improve the ability to move a virtual hand with a myoelectric controller. A tactile-only percept was chosen to avoid potential muscle activation associated with proprioceptive percepts, which could result in increased raw EMG magnitude resulting from electrical stimulation, regardless of iSens® noise rejection capabilities.

S01 controlled a right-sided virtual 3-dimensional hand, displayed on the same side as the residual limb on a monitor, using the 3 DOF ANN myoelectric controller. On the side of the monitor that corresponded to the sound limb, a left-sided virtual hand displayed a randomly chosen target posture, or a set of joint angle positions for each DOF [14, 16]. While the contralateral virtual hand remained stationary to present the target posture, the participant freely controlled the right-sided virtual hand via the 3 DOF myoelectric controller. The 3-dimensional hands were shown on a 2-dimensional monitor, so applying sensory feedback that varied with virtual arm position could have provided additional position information. The participant wore a socket and prosthesis during testing to replicate the socket pressure and prosthesis weight conditions, but the prosthesis itself did not move.

Within a trial limit of 30 s, the participant attempted to move the virtual hand to each target posture. A successful trial required holding the virtual hand within 15% of the target posture joint angles in all 3 DOFs for 1 consecutive second. In the condition with stimulation, pulse frequency increased from 0 to 50 Hz as virtual limb joint angles for all DOFs fell within 22.5% of the target posture, and frequency increased from 50 to 100 Hz when all joint angle DOFs fell within 15% of the target posture. Pulse width and amplitude remained constant at 250 μs and 0.3 mA, respectively. Outcome measures included time-to-target, or the total time to complete a successful trial, and path efficiency, calculated as the Euclidean distance between the starting position and final position per trial divided by distance traveled during the trial in joint angle space. Time-to-target and path efficiency were compared between with and without stimulation conditions with paired Wilcoxon signed-rank tests.

*Functional performance:* S01’s 3 DOF ANN controller was loaded to the Hub, which connected to an Android phone and DEKA LUKE arm. Pressure against prosthesis sensors drove median nerve C-FINE stimulation to evoke sensations in the thumb, index finger, and palm or dorsal hand (Supplementary Table S2, Additional File 1). Activated force sensors included the thumb, middle finger, and proximal palm. Exceeding sensor value thresholds resulted in increased pulse frequency across three discrete values to increase perceived intensity (Supplementary Table S2, Additional File 1). The middle finger sensor on the DEKA Luke arm was used due to the index finger sensor not responding during experimental sessions. To assess daily living functional performance, we implemented the Activities Measure for Upper Limb Amputees (AM-ULA) [53], repeating the test three times with and without stimulation. The AM-ULA is a validated measure in which a trained occupational therapist evaluates a prosthesis user’s ability to complete 18 tasks of daily living by scoring the user’s completion of sub-tasks, movement quality, speed, skillfulness, and independence. Following the validated protocol, AM-ULA summary scores were calculated by taking the mean overall score per task across repetitions, taking the mean across overall scores for all tasks, and multiplying by 10. Trials in which the prosthesis malfunctioned were removed from the analysis. The difference in AM-ULA scores between with and without stimulation conditions was compared to a minimum detectable change (MDC) of 4.4 for a 95% confidence interval [53]. S01 responded to five survey questions after each AM-ULA task during two repetitions per condition (Supplementary Table S3, Additional File 1). Survey responses regarding the participant’s perceived performance, confidence, difficulty performing the task, and frustration were compared between with and without stimulation conditions with right-tailed two sampled Wilcoxon signed-rank tests. The participant’s responses regarding the helpfulness of sensation were compared to a score of 0 with a right-tailed Wilcoxon signed-rank test.

### Statistical analysis

All data analyses were performed in MATLAB. The threshold for statistical significance was set to α = 0.05.

## Results

Implantation of the iSens system, an outpatient procedure, took 5 h for S01 and 5.25 h for S02. Recovery progressed on the expected timeline, and all incisions were healed at the two-week incision check. After iSens® implantation, neither participant experienced any changes in motor function or sensation in their residual limb. Neither participant experienced any changes in phantom pain or phantom sensations as a result of the surgery. Surgery-related pain for S01 and S02 lasted three and two days, respectively, before subsiding.

While this manuscript reports on the first 27 months post-implantation for S01, his implant remained stable beyond this period and his participation is ongoing. S02’s system was explanted after 15 weeks due to an infection of unknown origin. Pathology results indicated the infection resided at S02’s INC site and the posterolateral site in the upper arm, but the infection was not present in the anteromedial incision that housed the proximal median and ulnar nerve C-FINEs. Thus, the infection was unlikely to have influenced the sensory percepts or electrode stability measures on the two functional C-FINEs.

### The iSens® system successfully communicated wirelessly between the external Hub and implanted neural controller

**Fig. 3:**
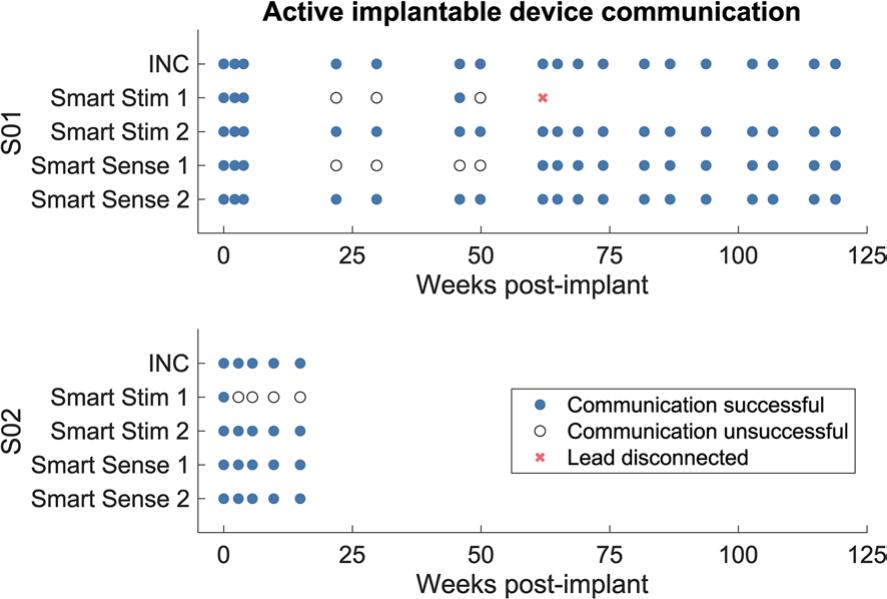
Active implantable device communication across time. Successful and unsuccessful communication at each encounter for S01 (top) and S02 (bottom). Wireless communication between the Hub and INC was confirmed by successful reception of BLE RSSI and battery level information by the Hub. Wired communication between the INC and each Smart Lead was confirmed by successful discovery of each Smart lead as shown in the Hub user interface. One Smart Stim Lead in S01 was disconnected and left unpowered during the revision surgery due to surgical risks associated with replacing the module

The INC communicated with the Hub during all encounters for both S01 and S02 (Fig. 3). Two of S01’s Smart Leads stopped communicating with the INC 22 weeks after the original surgery, except for one encounter in which the Smart Stim successfully communicated with the INC during week 46. After the revision surgery, S01’s INC successfully connected to the remaining three Smart Leads during all encounters. During intraoperative testing for S02’s iSens® implant, the INC communicated successfully with all four Smart Leads. During subsequent visits, S02’s INC reliably communicated with three Smart Leads during all encounters but did not discover one of the Smart Stim Leads. Bench testing of S02’s iSens® system that was recovered during his explant revealed that a loose set screw in the INC inline bal-seal connector caused the communication failure, emphasizing the importance of stable lead connections.

### Peripheral nerve stimulation was chronically stable in a wirelessly connected system

**Fig. 4:**
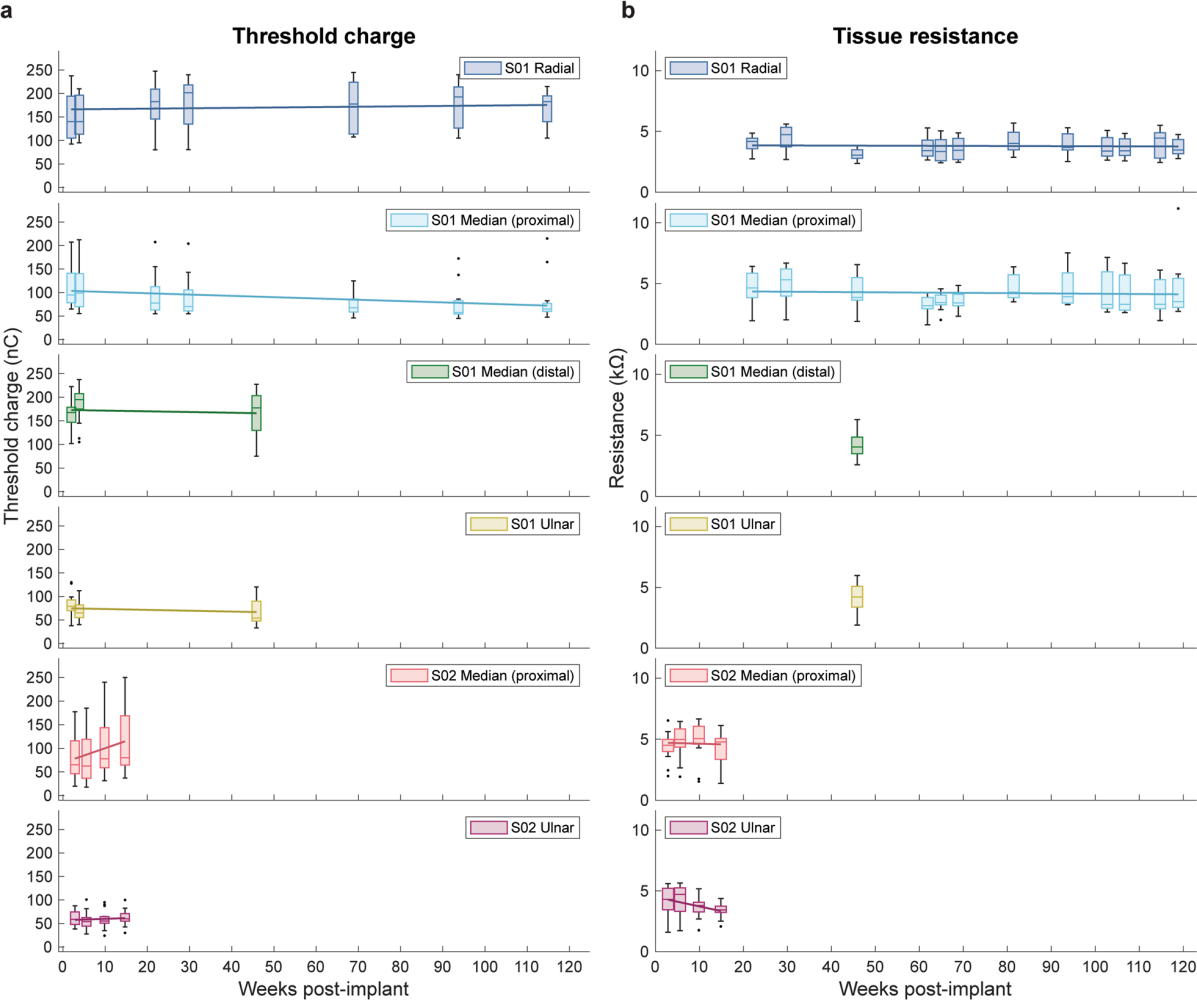
Threshold charge and tissue resistance across time. **a** Perceptual threshold charge and **b** tissue resistance across time for each C-FINE. Box charts represent the distribution of threshold and resistance values across all contacts per C-FINE within each visit, and solid lines represent the fit linear regression model across all time points

Stimulation charge at sensory detection threshold remained stable across 46 and 115 weeks for C-FINE contacts in S01’s two Smart Stim Leads (Fig. 4a). Although S01 underwent a revision surgery to replace bifurcated leads connecting the Smart Leads to the INC, the surgery did not involve updating the C-FINEs, so we did not expect the revision surgery to impact S01’s sensory perceptions or electrode reliability for the accessible C-FINEs. Mean thresholds for eliciting sensory perception for S01 and S02 were 123.9 ± 59.9 nC and 77.18 ± 49.1 nC, respectively, across all C-FINEs. For each C-FINE, S01’s mean perceptual thresholds were 170.6 ± 48.3 nC, 91.4 ± 43.5 nC, 170.8 ± 39.8 nC, and 71.9 ± 25.8 nC for the radial nerve, proximal median, distal median, and ulnar nerve C-FINEs, respectively. S01’s radial nerve C-FINE threshold charge increased slightly, but not significantly, at 0.082 nC/week (F-test, p = 0.55). Threshold charge for ulnar, distal median, and proximal median nerve C-FINEs changed at rates of − 0.18 nC/week, − 0.15 nC/week, and − 0.28 nC/week, respectively, indicating that the stimulation charge required to reliably elicit sensation decreased over time. This decrease in threshold charge was not significantly different from a constant regression model for S01’s ulnar and distal median nerve C-FINEs (F-test, p = 0.36 and p = 0.63, respectively) but was significantly different from the constant regression model for the proximal median nerve C-FINE (F-test, R^2^ = 0.069, p = 0.0091). Mean perceptual thresholds for each of S02’s C-FINEs were 94.7 ± 62.5 nC for the proximal median nerve and 59.3 ± 17.4 nC for the ulnar nerve. S02’s ulnar and proximal median nerve C-FINEs threshold charge increased slightly, but not significantly, at 0.31 nC/week and 3.08 nC/week, respectively (F-test, p = 0.54 and p = 0.086, respectively) (Fig. 4a).

Tissue resistance for functional contacts across all time points and for both study participants ranged between 1.4 kΩ to 11.2 kΩ, with a mean and standard deviation of 4.1 ± 1.2 kΩ (n = 85 contacts, with multiple measures per contact). Five of S01’s contacts were excluded from the analysis due to resistance values exceeding 15 kΩ, which were interpreted as inconsistent connections between the C-FINE contact and Smart Stim rather than true changes in tissue resistance. Although we were unable to assess S01’s distal median and ulnar nerve C-FINE tissue resistances across time prior to powering down the corresponding Smart Stim Lead during the revision surgery, a single measurement assessed at 46 weeks post-implant demonstrated that the tissue resistance for these C-FINEs also fell within the specified range. Tissue resistance remained stable across time or decreased for fully operational C-FINEs in S01 and S02 (n = 4 cuffs, totaling 56 contacts) (Fig. 4b). C-FINE tissue resistance linear regression models for S01’s radial and proximal median C-FINEs, as well as S02’s proximal median C-FINE, were not significantly different from constant models (F-test, p = 0.68, p = 0.54, and p = 0.79, respectively). S02’s ulnar C-FINE tissue resistance significantly decreased at a rate of -0.078 nC/week (F-test, R^2^ = 0.11, p = 0.0089).

### iSens® stimulating modules evoked somatosensory percepts spanning the hand

**Fig. 5:**
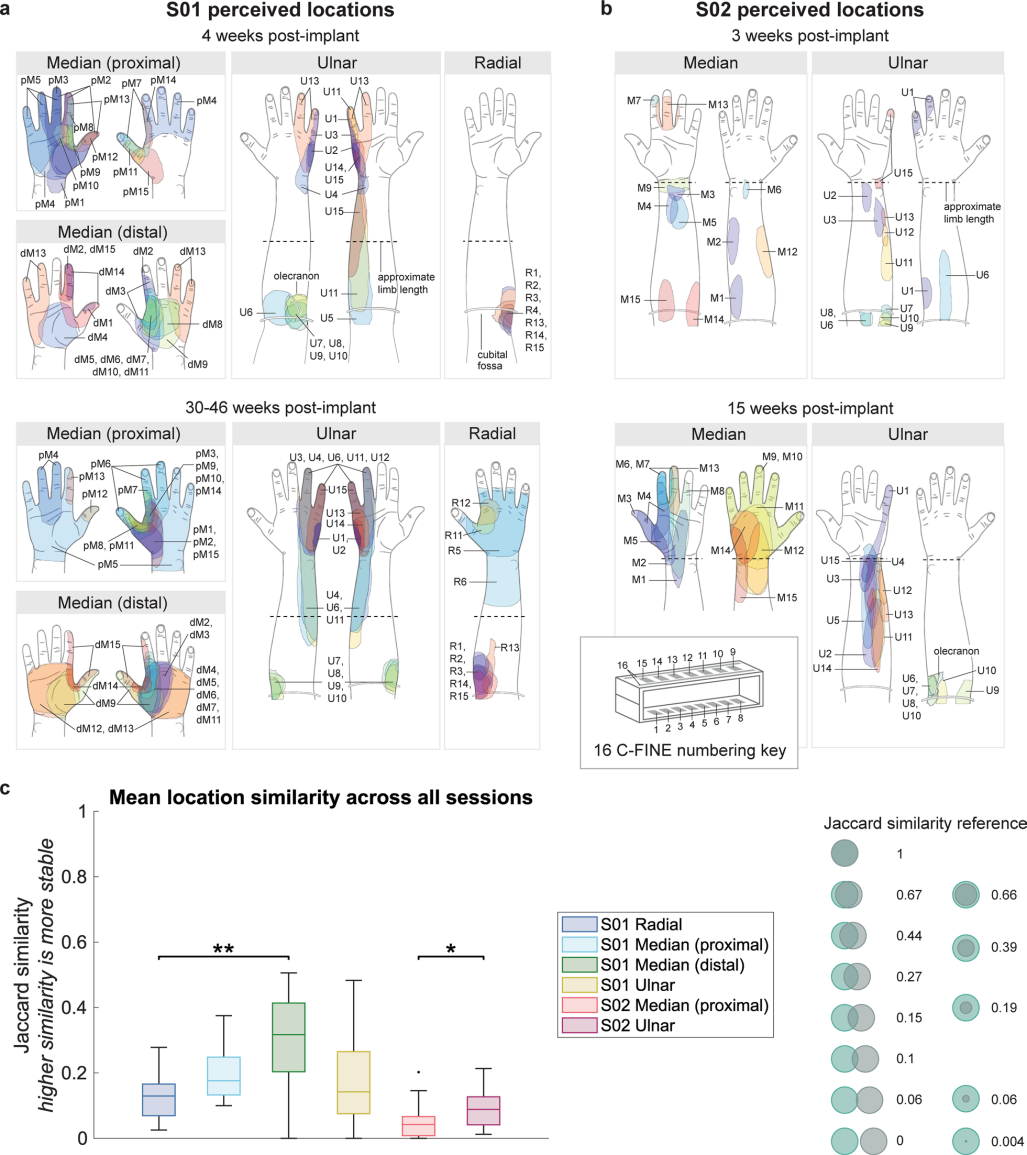
iSens®-elicited sensory locations. Somatosensory locations, excluding proprioception-only percepts, elicited with iSens® during two testing sessions for **a** S01 and **b** S02. All locations were drawn at threshold perception. **c** Location similarity across all combinations of experimental sessions per C-FINE contact, grouped by C-FINE. A higher Jaccard similarity indicates more instances of overlapped pixels between location drawings from multiple sessions, suggesting stable locations. A Jaccard similarity of 1 represents two perfectly overlapped location drawings, and a Jaccard similarity of 0 represents no overlap. Jaccard similarities greater than 0 but less than 1 indicate that the two locations are in similar areas but vary in terms of the center position and area size. Significance: * represents p < 0.05, and ** represents p < 0.01

iSens®-evoked percepts were located on the front and back of the hand, the wrist, and the residual limb for S01 and S02 (Fig. 5a, b). Perceived location stability per C-FINE contact was estimated by the average Jaccard similarity between location drawings across all experimental session combinations (Fig. 5c). Any Jaccard similarity greater than zero indicates that two sensory areas overlap in the same general region, but a Jaccard similarity less than 1 indicates that the size and boundaries have some variation in location size or position between sessions. Mean Jaccard similarity values across all contacts for S01’s radial nerve C-FINE, proximal and distal median nerve C-FINEs, and ulnar nerve C-FINE were 0.1 ± 0.07, 0.2 ± 0.08, 0.3 ± 0.2, and 0.2 ± 0.1. S02’s mean Jaccard similarities were 0.06 ± 0.06 for the median nerve C-FINE and 0.09 ± 0.06 for the ulnar nerve C-FINE. As mean Jaccard similarities across all C-FINEs were greater than 0 but less than 0.5, each C-FINE contact’s evoked locations demonstrated a mixture of maintaining location overlap as well as shifting to different locations between sessions, with certain C-FINE contacts showing heightened location stability and others shifting consistently between sessions (see Supplementary Fig. S1, Additional File 1 for examples). S01’s perceived locations evoked from the distal median nerve C-FINE showed significantly increased Jaccard similarity compared to radial nerve C-FINE locations, indicating that the distal median nerve C-FINE produced more stable locations compared to the radial nerve C-FINE (Chi-squared test, p = 0.0068). No significant stability differences were found between the proximal median nerve or ulnar nerve C-FINE locations. S02’s ulnar nerve C-FINE locations demonstrated significantly higher Jaccard similarities compared to median nerve C-FINE locations, showing that locations evoked by the median nerve changed more across sessions (Wilcoxon rank-sum test, p = 0.038). Overall, S02’s perceived locations demonstrated lower mean Jaccard similarity coefficients and less stability compared to S01’s reported locations for each C-FINE.

**Fig. 6:**
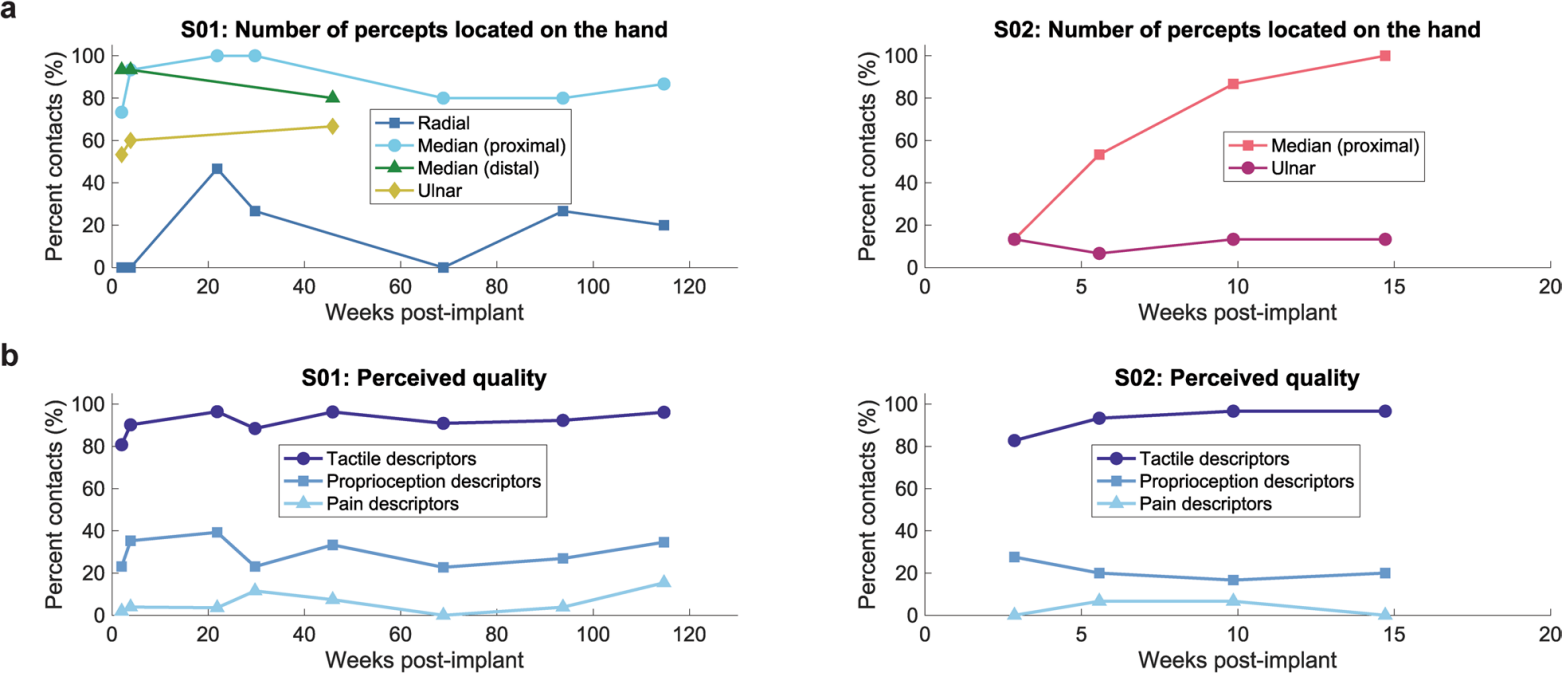
Percent of percepts reported on the hand and categorized quality descriptors across time. **a** Percent of contacts per C-FINE evoking percepts located in the hand, distal to the wrist, across time for S01 (left) and S02 (right), excluding proprioception-only percepts. **b** Percent of all C-FINE contacts evoking tactile, proprioceptive, and pain quality descriptors as reported by S01 (left) and S02 (right) across time. Participants could report an unlimited number of quality descriptors, so the total percentage reported across all quality descriptor categories does not sum to 100%

Contacts on S01’s radial, proximal median, distal median, and ulnar nerve C-FINEs and S02’s ulnar nerve C-FINE demonstrated similar percentages of sensory locations in the hand across time, as the logistic regression models did not significantly differ from a constant model (Chi-squared test, p = 0.42, p = 0.45, p = 0.29, p = 0.51, and p = 0.81, respectively) (Fig. 6a, left). Although S02 reported locations mostly in the forearm during his first session, he experienced more sensory locations shifting to the hand region across time (Chi-squared test, p << 0.001) (Fig. 6a, right). Grouped across all time points, S01 reported significantly more percepts in the hand when stimulating through the proximal and distal median nerve C-FINEs compared to the radial nerve C-FINE, but no significant differences were observed when comparing S01’s ulnar nerve C-FINE to the other three C-FINEs or between the two median nerve C-FINEs (Chi-squared test, p = 0.0012). S02 significantly more often reported percepts on the hand when stimulating through the median nerve C-FINE compared to the ulnar nerve C-FINE (Chi-squared test, p = 0.046). Across all sessions, 17 ± 18% radial nerve contacts, 88 ± 10% proximal median nerve contacts, 89 ± 8% distal median nerve contacts, and 60 ± 7% ulnar nerve contacts in S01 evoked percepts on the hand (mean ± standard deviation). For S02, 63 ± 39% median nerve contacts and 12 ± 3% ulnar nerve contacts evoked hand percepts.

Quality descriptors associated with sensory percepts evoked by C-FINE contacts were categorized into tactile, proprioceptive, and pain descriptors (Supplementary Table S4, Additional File 1). S01’s frequency of reporting tactile, proprioceptive, and pain percepts did not significantly change across time (Chi-squared test, p = 0.070, p = 0.96, and p = 0.12, respectively) (Fig. 6b). S02’s frequency of reporting proprioceptive and pain descriptors remained stable across time (Chi-squared test, p = 0.50 and p = 0.79, respectively), but the percent of contacts associated with tactile qualities increased across time (Chi-squared test, p = 0.044). Grouped across all time points, both participants more often reported tactile percepts compared to pain percepts, but proprioception reporting frequency showed no differences compared to tactile or pain percepts (Chi-squared test, S01: p << 0.001, S02: p = 0.0068). Grouped across all sessions, S01 reported tactile percepts at 91 ± 5% of contacts, proprioception at 30 ± 7% of contacts, and pain at 6 ± 5% of contacts. S02 reported tactile percepts at 92 ± 7% of contacts, proprioception at 21 ± 5% of contacts, and pain at 3 ± 4% of contacts.

The majority of reported descriptor percentages remained stable across time (Supplementary Fig. S2, Additional File 1). Three descriptors reported by S01 and six descriptors reported by S02, however, demonstrated significant changes in the percent of contacts associated with each descriptor across time. S01’s reporting of “tickling” and “tight” significantly increased (Chi-squared test, p << 0.001 and p = 0.0066, respectively), and his reporting of “contraction” significantly decreased across time (Chi-squared test, p = 0.0015). S02’s reporting of “vibration,” “tingle,” “electrical,” and “pulsing” significantly increased across time (Chi-squared test, p = 0.0048, p = 0.032, p << 0.001, and p = 0.018, respectively). S02’s reporting of “buzzing” and “tight” decreased across time (Chi-squared test, p << 0.001 and p = 0.0095, respectively). Both S01 and S02 reported the word “vibration” most often across all sessions compared to all other descriptors, 67% and 66% of the time, respectively (Supplementary Table S4, Additional File 1). Second most frequently reported descriptors were “pressure” for S01 (31%) and “electrical” for S02 (45%).

### iSens® myoelectric sensing modules demonstrated stable channel crosstalk across time

S01 and S02 demonstrated responsive EMG channels, classified by modulating EMG amplitudes during voluntary residual limb movements (Supplementary Fig. S3, Additional File 1). After S01’s revision surgery, Smart Sense communication was restored to enable EMG recording from 16 EMG channels during all subsequent sessions (Supplementary Fig. S3a, Additional File 1). Two of S01’s EMG channels, both in the same TIM, produced low-amplitude, noise-only recordings starting at week 65 and then later at week 115 for the second channel. We hypothesize that an unstable connection at more proximal point along the circuity may have affected the signal quality in the two unresponsive EMG channels. S02 demonstrated stable recording quality from 16 EMG channels during all three experimental sessions across 10 weeks (Supplementary Fig. S3b, Additional File 1).

**Fig. 7:**
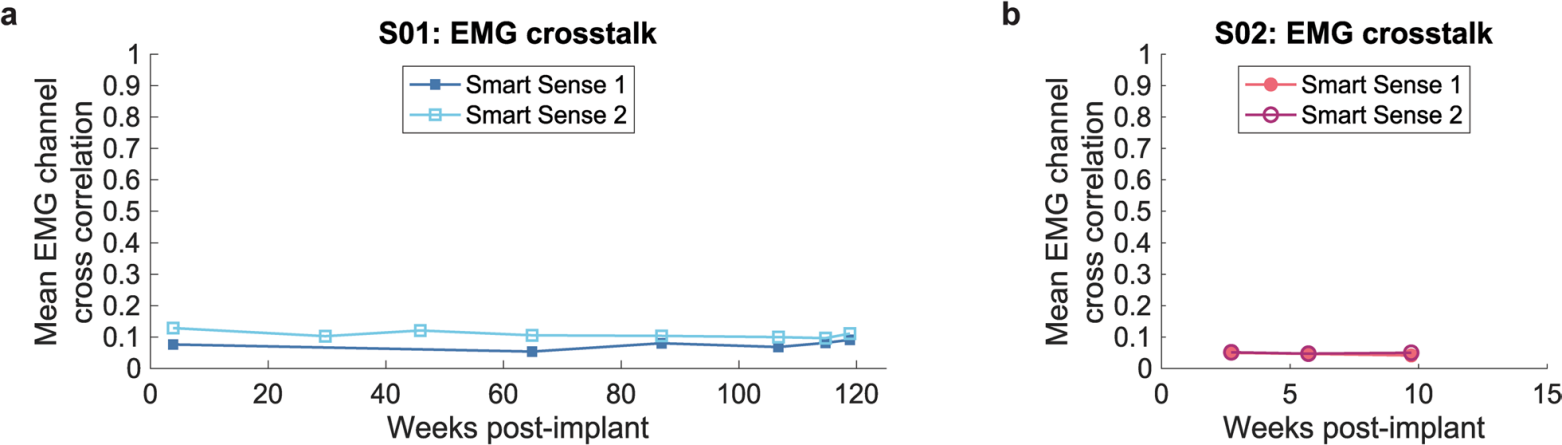
EMG channel crosstalk across time. Mean EMG channel cross correlation per Smart Sense Lead, from eight channels, across time in **a** S01 and **b** S02

Averaged across Smart Sense channel combinations, S01 and S02’s Smart Sense modules produced stable levels of low crosstalk across time as demonstrated by constant or decreasing mean channel cross correlations (Fig. 7b). Linear regression slopes of mean channel cross correlation values across time for both of S01’s Smart Sense Leads and one of S02’s Smart Sense Leads were not significantly different from constant models, indicating similar levels of channel crosstalk at each experimental session (F-test, p = 0.52, p = 0.07, and p = 0.83, respectively). One of S02’s Smart Sense Leads showed a slight but significant decreasing cross correlation trend, indicating a slight improvement in channel crosstalk over time (F-test, R^2^ = 1.0, p = 0.015). Smart Sense modules demonstrated low crosstalk, below 0.10, between the majority of channel combinations (Supplementary Fig. S4, Additional File 1). S01’s supinator and extensor carpi radialis longus muscles, as well as S02’s pronator and flexor pollicus longus muscles, demonstrated elevated channel crosstalk, exceeding 0.4, compared to other channel combinations. S01 and S02 also showed slightly elevated crosstalk between multiple channels implanted in the same muscles, including extensor digitorum communis and flexor digitorum superficialis muscles, which was anticipated due to close channel proximity and likelihood of the muscles showing similar activation at multiple recording sites. S01 demonstrated elevated crosstalk across time between two channels implanted in the flexor digitorum superficialis muscle as well as between extensor carpi radialis and extensor carpi ulnaris muscles during week 119 post-implant, which could indicate that the electrodes recorded higher levels of common mode noise (Supplementary Fig. S4, Additional File 1). However, the elevated channel crosstalk between these two channel combinations in S01, out of a total of 56 channel combinations, did not affect mean crosstalk regression results that showed stable overall Smart Sense crosstalk.

### iSens® myoelectric sensing modules rejected stimulation artifacts

The goal of the iSens® Smart Sense module was to reliably record EMG activity associated with intended hand and arm movements for prosthesis control without being disrupted by ongoing nerve stimulation for sensation delivered nearby.

**Fig. 8:**
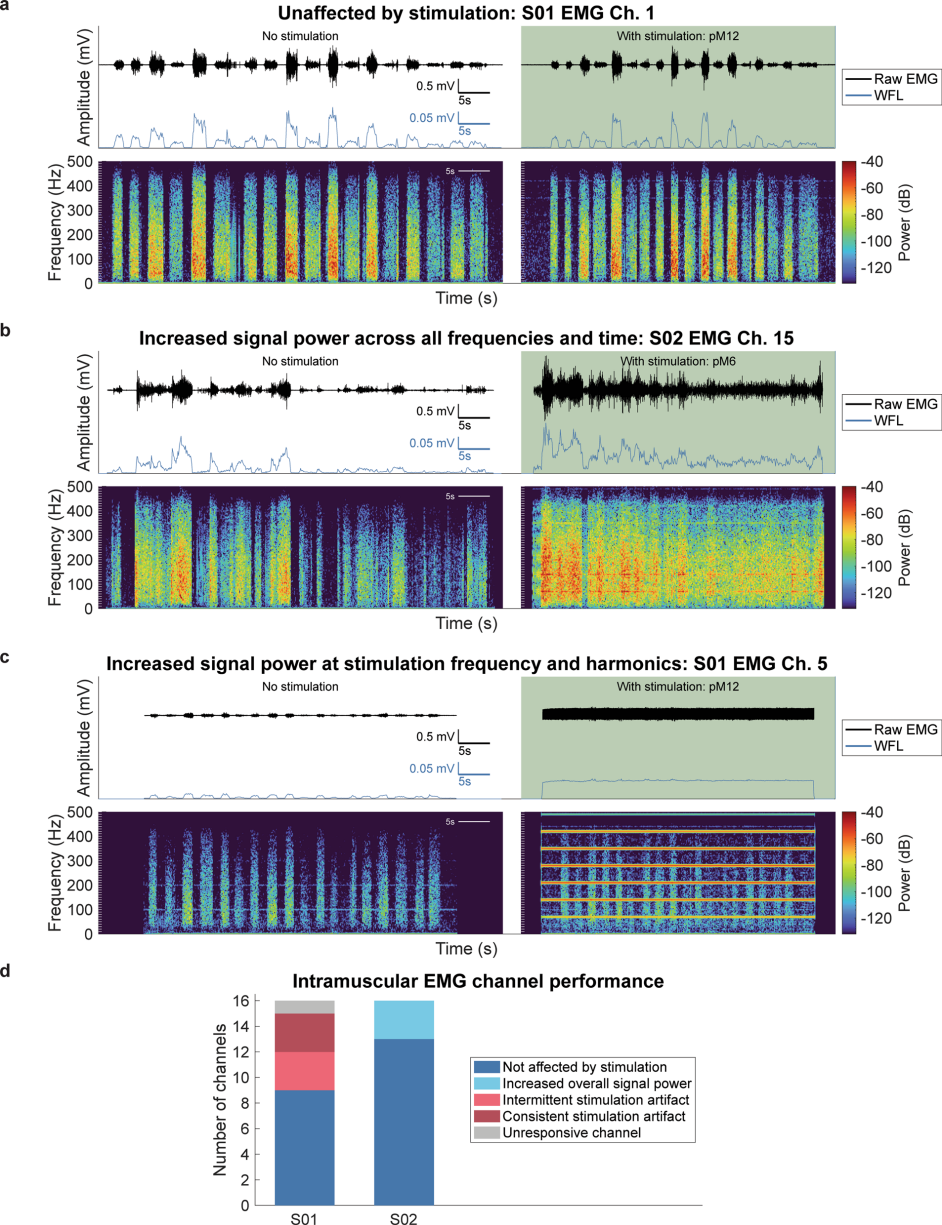
Intramuscular EMG channel performance. Examples of raw EMG and computed WFL recorded while participants move to 18 postures, with and without 70 Hz single contact stimulation via the proximal median (pM) nerve C-FINEs. **a** Example EMG channel that displayed low noise and clear amplitude modulations during rest and movement phases, regardless of stimulation. **b** Example EMG channel that showed increased EMG signal power with stimulation throughout all moments in time and at all frequencies. **c** Example EMG channel that showed increased noise at the stimulation frequency and harmonics through the trial. **d** Categorization of intramuscular EMG recording performance for each participant

Without stimulation, 15 and 16 EMG channels out of the total 16 channels in S01 and S02’s systems, respectively, demonstrated high quality recordings, as indicated by clear increases in amplitude during attempted movements and low signal amplitude during rest (Fig. 8a). Post-hoc calculated WFL correlated well with increases in raw EMG amplitude, supporting the choice of WFL to represent movement intent with less impact from offsets (Fig. 8a–c). With active stimulation, 9 and 13 EMG channels for S01 and S02, respectively, continued to demonstrate high quality EMG recordings (Fig. 8a, d). Negligible noise was observed at the stimulation frequency and harmonics on these channels. Thus, these channels were not affected by stimulation and could record viable EMG signals for myoelectric control during concurrent sensory feedback.

Three of S02’s EMG channels showed increased signal power across all frequencies and throughout both rest and movement phases during a single trial with stimulation via one of the two tested C-FINE contacts (Fig. 8b, d; see Supplementary Fig. S5a, Additional File 1 for mean ratio-of-overall-power-increased per EMG channel). This observation could indicate stimulation of motor efferents, a voluntary change in contraction, or presence of stimulation artifact. These three EMG channels recorded activity from pronator teres, flexor pollicus longus, and flexor carpi radialis muscles, which could be activated by stimulation of efferents via the median nerve C-FINE. Power at the stimulation frequency (70 Hz) and harmonics was slightly elevated relative to neighboring frequencies, which could indicate activation of efferents or stimulation artifact (Fig. 8b). Prior to recording EMG, S02 experienced visible “pronation” when stimulating through this same C-FINE contact at higher pulse amplitudes, supporting the idea that motor efferent activation caused the elevated signal power. Alternatively, the increase in EMG signal power across frequencies and time could indicate a voluntary change in muscle tension or movement strategy, in which S02 did not relax completely during rest periods. In contrast, none of S01’s EMG channels showed increased overall signal power during stimulation throughout recordings.

Three of S01’s EMG channels experienced consistent disruptions due to stimulation artifact, as evidenced by increases in signal power within narrow bands at the stimulation frequency and harmonics during stimulation through all three tested C-FINE contacts (Fig. 8c, d). Frequency spectra demonstrated that muscle activity was still being recorded during active stimulation at these three channels, but the signals contained overlaid artifacts at the stimulation frequency and harmonics (Fig. 8c). An additional three of S01’s EMG channels, all within the same TIM, recorded high noise at the stimulation frequency during a single EMG recording trial but negligible artifact during the other two trials (Fig. 8d). We palpated the region of S01’s arm where the corresponding Smart Sense was located and noted intermittent high levels of noise in the EMG time domain signal, indicating a potential loose connection at one of the connectors between the Smart Sense and TIM. The six EMG channels in S01’s system that experienced artifacts at the stimulation frequency (see Supplementary Fig. S5b, Additional File 1 for mean ratio-of-frequency-power-increased per EMG channel) also showed an increase in power at the Smart Sense wired packet rate (100 Hz), suggesting poor common mode rejection that we hypothesize was caused by connector issues and not the TIMs or Smart Sense modules themselves (Fig. 8c).

At the time of EMG stimulation artifact testing, one EMG channel in S01’s system demonstrated a noise-only recording (Fig. 8d). None of S02’s 16 EMG channels demonstrated the same narrow bands of increased signal power at the stimulation frequency that was experienced in six of S01’s EMG channels. S01’s ten EMG channels that were unaffected by stimulation were used as ANN inputs to train and develop a 3 DOF myoelectric controller for real-time control, as discussed in subsequent sections.

### Participants successfully controlled a virtual hand in 3 DOF with simultaneous stimulation by using the iSens® system

**Fig. 9:**
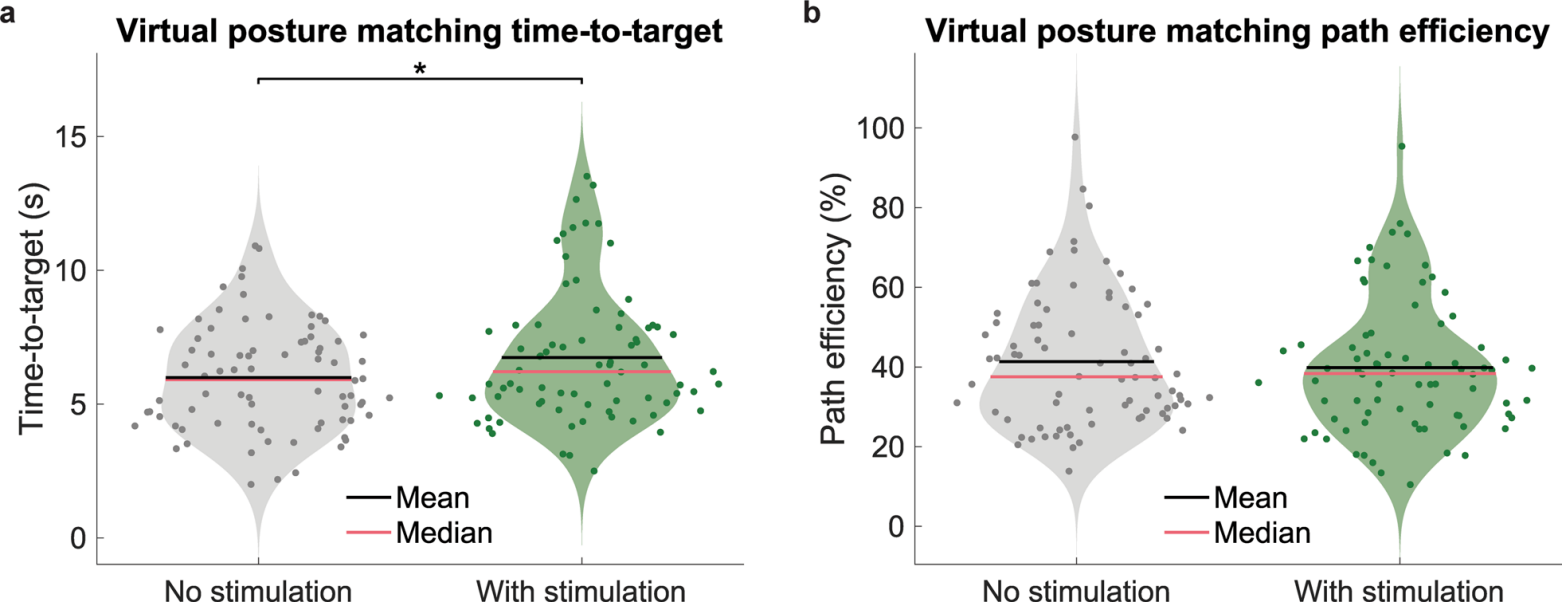
Virtual environment myoelectric control posture matching using iSens® and a 3 DOF ANN controller. **a** S01’s time-to-target to match postures presented on a monitor screen, with and without stimulation. Each target posture was presented three times for each condition. **b** S01’s path efficiency while attempting to move to each posture, with and without stimulation

S01 successfully matched 100% of all target postures without stimulation and 99.6% of all target positions with stimulation within the 30 s trial limit (78 target postures per set, 3 sets per stimulation condition). Time-to-target significantly increased by approximately 0.3 s/target during trials with stimulation (Wilcoxon signed-rank, p = 0.049) (Fig. 9a). Median time-to-target was 6.2 ± 2.5 s/target with stimulation and 5.9 ± 2.0 s/target without stimulation. However, path efficiency was not affected by stimulation, as demonstrated by the lack of significant difference between the with and without stimulation conditions (Wilcoxon signed-rank, p = 0.52) (Fig. 9b). Median path efficiency was 38 ± 17% with stimulation and 38 ± 17% without stimulation. S01 reported a change in concentration and movement strategy between stimulation conditions. He noted, “Stim was good with letting me know I was close to [the target position] and everything. … without stim, I probably concentrated a little bit more on the posturing of the muscles for getting [the virtual hand] into the position. … With the stim, I was trying to focus on the muscles, but stim also. It was like, when I feel [the stimulation] real light, I know I’m getting close, so then I’m trying to concentrate on that a little more.” When asked about if he felt any difference in difficulty completing the task with stimulation, S01 responded, “Not really any [difference in] difficulty …,” despite the greater trial time.

### Stimulated sensation during functional performance was perceived as helpful

**Fig. 10:**
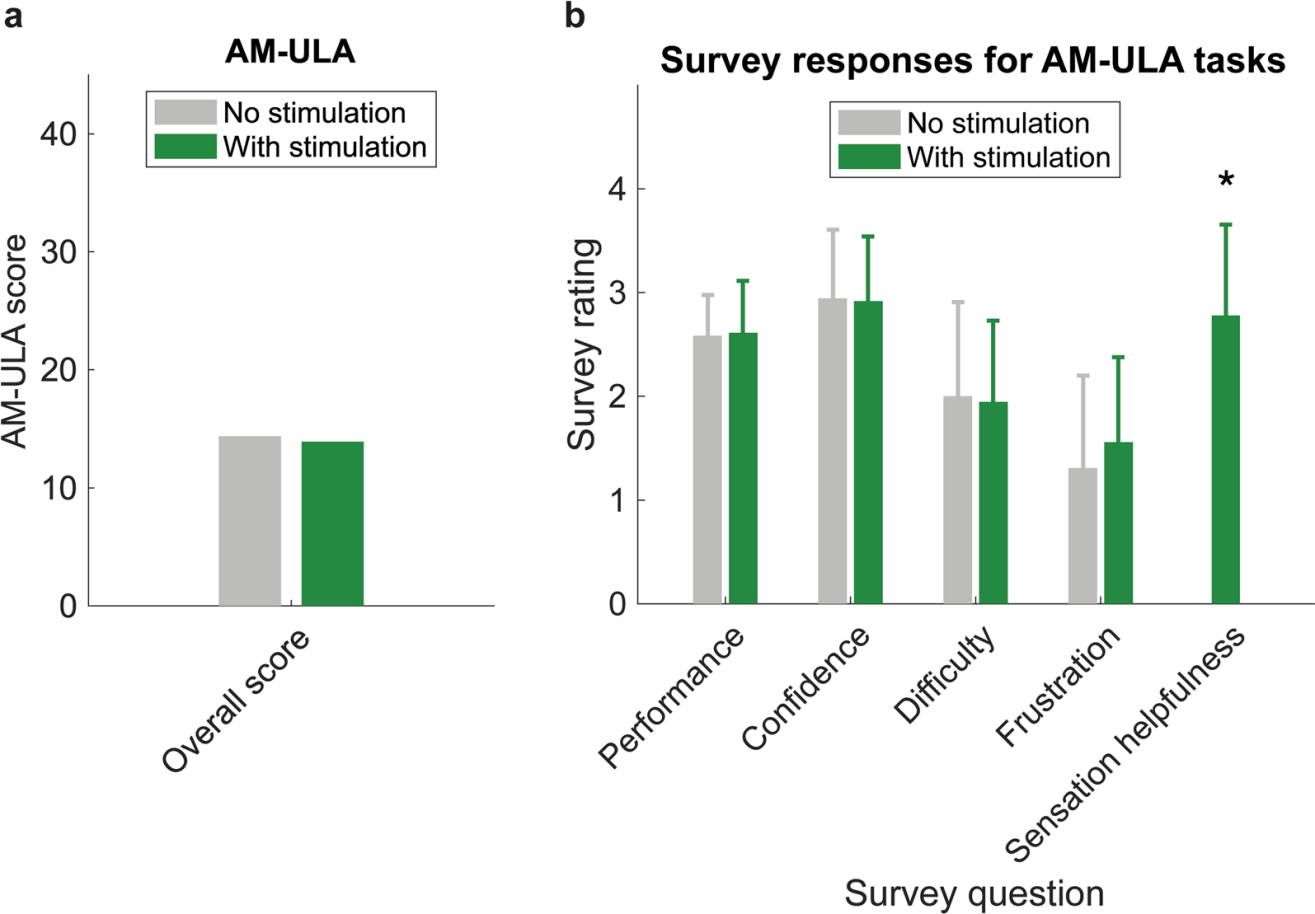
Activities of daily living functional performance using iSens® and an advanced prosthesis with sensors. **a** AM-ULA summary score for each condition. The AM-ULA was repeated three times per condition, with and without stimulation. **b** Survey rating mean and standard deviation for survey questions reported after each task in the AM-ULA, with and without stimulation

The same 3 DOF myoelectric controller was used during both AM-ULA experimental sessions, without any retraining during the second session. Mean AM-ULA scores were slightly higher without stimulated sensation (14.4) than with stimulated sensation (13.9) (Fig. 10a), but this difference did not exceed the MDC (MDC = 4.4) and thus does not represent a meaningful difference at a 95% confidence interval [53]. Therefore, stimulation did not affect functional performance during the AM-ULA. Averaged across all tasks and two repetitions of the AM-ULA, S01 rated the extent to which sensation helped him perform each task as 2.8 ± 0.6 out of a maximum of 4 (Fig. 10b; Supplementary Table S3, Additional File 1). S01’s ratings demonstrated a significant positive effect of sensation on perceived task performance (Wilcoxon signed-rank test, p << 0.001). Ratings for perceived performance, confidence, task difficulty, and frustration across all AM-ULA tasks were not statistically different with sensation compared to no sensation.

Regarding perceived location stability across the two AM-ULA experimental sessions, thumb and index finger sensations remained in the same perceived digit areas between both lab visits. However, the palm sensation experienced in the first session changed to the dorsal hand during the second session (Supplementary Fig. S6, Additional File 1).

## Discussion

### Impacts of fully implantable neural interfaces

To the authors’ knowledge, this is the first clinical report of a wirelessly connected neuroprosthetic device to restore somatosensory feedback and myoelectric control for limb loss. By integrating the INC, the iSens® system avoids the need to carry around an external neural interface processor, as required by percutaneous upper extremity restoration systems [35]. Placement of the Hub near the residual limb supported stable BLE communication for EMG signal streaming and sending stimulation commands, showing that the Hub did not need to be placed directly over the INC to maintain communication. Exclusion of percutaneous leads in the iSens® system’s design eliminated the need for participants to consistently clean and protect percutaneous exit sites [54]. Subjects implanted with iSens® also received an increased number of stimulating and recording channels compared to previous systems with FINEs and TIMs, as limits on the density of conductors passing across the skin reduces the total number of electrodes in percutaneous systems [14, 28].

### iSens® components exhibited chronic stability required for long-term clinical use

We have shown a stable, high channel-count extraneural interface for somatosensory and hand function restoration. iSens® sensory detection thresholds and tissue resistance values across time demonstrate stable trends, mirroring prior literature regarding extraneural cuff electrodes [28, 31, 55, 56]. After initially implanting nerve cuff electrodes, an increase in threshold charge is expected due to tissue inflammation from the surgical procedures. As the tissue heals, encapsulation tissue forms and secures cuff electrode positions around peripheral nerves. After approximately 20 weeks, threshold charge is expected to decrease and stabilize [28, 31, 55, 56]. Extraneural cuff electrodes have been implanted in humans and utilized for up to 12 years, further supporting longevity for stimulated somatosensation [28].

iSens® Smart Sense modules demonstrated stable channel crosstalk, which is important for maintaining high DOF myoelectric controller performance long-term. Channel crosstalk is an estimate of signal independence, and therefore, decreasing channel crosstalk allows for more unique muscle signals to distinguish between intended movements. Mean channel cross correlation found with iSens® Smart Sense modules exceeded cross correlation values reported in one study evaluating TIM EMG and surface EMG crosstalk [14] but demonstrated comparable or improved correlation values compared to other reports of surface EMG studies [57–59]. Compared to previous upper extremity studies estimating implanted EMG crosstalk, iSens® Smart Sense modules demonstrated slightly increased mean channel crosstalk [14, 60, 61]. We hypothesize that the increase in total channels, as well as the number of EMG channels implanted in identical muscles, contributed to the elevated crosstalk values shown in Smart Sense Leads. iSens® EMG channel independence across 10 channels enabled participant S01 to successfully control virtual and prosthetic limbs in 3 DOFs. Additionally, the participant did not need to retrain the controller during subsequent experimental sessions, spanning 12 weeks after originally training the controller, which is similar to previous reports of TIM myoelectric controllers [14, 16]. This is a significant advantage over surface EMG systems requiring at least daily retraining sessions.

Overall, iSens® Smart Sense Leads and TIMs continued to record high quality signals to support real-time myoelectric control, but we hypothesize that connector mechanical integrity issues contributed to the lower quality signals demonstrated in some of S01’s EMG channels. S01’s change in responsive EMG channels, intermittent noise when physically palpating near the Smart Sense implant area, and stimulation artifact presence in six of S01’s EMG channels indicate potential changes in TIM-Smart Sense connector stability, emphasizing the need for further connector mechanical testing and development. Previous groups have investigated algorithms to remove stimulation artifacts in the raw EMG signal, prior to calculating EMG features [62], which could be implemented in a new iteration of the Smart Sense modules to account for poor common mode rejection observed in some of the channels. Updating the placement of bifurcated leads more laterally and away from the axilla during S01’s revision surgery and during S02’s implant surgery likely reduced mechanical stress on the leads, which should be implemented for future surgeries. Despite the connector stability issues, S01’s Smart Sense modules continued to record reliable EMG signals from the remaining EMG channels to support use of a 3 DOF myoelectric controller in real-time with simultaneous stimulation for sensory feedback. Additionally, S02 demonstrated 16 responsive EMG channels at all experimental sessions in addition to successful stimulation artifact removal.

Regarding the overall system’s longevity, wireless BLE communication between the INC and Hub remained stable across time for both participants, supporting chronic use of fully implanted systems. S01 and S02 experienced issues with wired communication to two and one Smart Leads, respectively, but the modular design allowed the rest of the system to function as expected. The ability to ignore or include implanted components as well as customize the placement and total number of electrodes supports continuous system functionality and subject participation despite individual component failures. Stimulating through two 16-channel C-FINEs for both participants enabled tactile percepts on the hand that could be combined with sensor locations on upper extremity prostheses. Recording from 10 EMG channels enabled the development of a reliable 3-DOF ANN controller for use in the virtual environment and with a prosthesis for functional testing across two experimental sessions.

The infection that led to S02’s explant highlights the need to reassess surgical preparation procedures prior to iSens® implantation. Concern for infection is appropriate due to many factors, including the length of surgery and the patient’s compromised limb area from the prior injuries and surgeries. Local infections typically necessitate full explant of the system, which is challenging and frustrating for the patient. In response, we modified perioperative management by adopting a similar protocol used in total joint arthroplasty, including antimicrobial prophylaxis and preoperative antimicrobial baths [63].

### iSens®-evoked somatosensory percepts demonstrated stable qualities and a combination of stable and shifting locations

Similar to previous implanted nerve stimulation studies, iSens®-evoked somatosensory percept locations spanned various hand locations [17, 28, 32, 33]. Tactile percepts evoked on the hand were congruent to sensor locations on a prosthesis, which has been shown to reduce cognitive load when using a prosthesis with sensory feedback [27]. Perceived locations evoked by individual C-FINE contacts with the iSens® system showed a combination of stable and shifting percepts between experimental sessions. When stimulating through implanted nerve electrodes during laboratory experimental sessions, previous groups reported relatively stable perceived locations from single-contact stimulation, with exceptions for a subset of contacts that showed changing locations across sessions [28, 32, 33]. Interestingly, S02’s median nerve shifting location percepts from the residual limb to the hand across time mirrored similar trends reported from both upper and lower limb sensory restoration systems [32, 64]. We hypothesize, in agreement with previous work [32, 64], that S02’s naïve experience to peripheral nerve stimulation and likely plastic changes within the somatosensory pathway after his amputation contributed to initially feeling more percepts in the residual limb, which later shifted to the hand. Previous studies revealed plasticity after upper extremity amputations that resulted in cortical representations of the missing hand correlating with touch to other body areas, such as the face, contralateral limb, and adjacent areas [65, 66]. Applying sensory feedback to the missing limb through targeted reinnervation resulted in receptive field changes that resembled pre-amputation cortical representations, supporting our hypothesis [67]. In contrast to infrequent nerve stimulation in laboratory settings, at-home trials testing sensory-enabled prosthetic hands used for daily living demonstrated location percepts that maintained high position stability when paired with sensors at different hand locations [68] or became increasingly aligned with prosthesis sensors in similar hand locations across study durations [36].

S01 and S02 reported consistent levels of tactile, proprioceptive, and pain descriptors across experimental sessions. Tactile and proprioceptive percepts can convey touch and movement feedback to prosthesis users. Individual quality descriptor variation between S01 and S02 may indicate plasticity in sensory perception. S02 had not participated in any prior sensory stimulation experiments. His total experience with stimulated somatosensory percepts summed to 9 days within 15 weeks in a laboratory setting. In contrast, S01 experienced stimulated somatosensation for 8 years as a participant in a prior study with implanted nerve electrodes before being implanted with the iSens® system. S01 also had prior experience with home use of a sensory-enabled prosthesis with electrically activated touch feedback, in which his reports of perceived “naturalness” within and across days increased during a 3-month at-home study [36].

### The iSens® system enabled simultaneous nerve stimulation and myoelectric recording to support bidirectional control in a non-percutaneous interface

S01’s 3 DOF controller time-to-target in the virtual environment matched previously published trial durations for myoelectric controllers with multiple DOFs [14, 16, 69–71]. We speculate that the slight increase in trial duration with stimulated sensations occurred due to a change in allocation of attentional resources while learning to associate tactile sensation intensity at the index finger with limb position. The participant used both visual and tactile sensory feedback modalities during the test to estimate virtual hand position. Tactile sensory feedback has provided sufficient information for identifying hand postures, and therefore, tactile sensation was implemented in place of proprioception to avoid involuntary muscle contractions [72]. It has been shown that multiple task-informative sensory modalities, such as touch and vision, compete for shared attentional resources [73]. S01’s index finger tactile sensation did not match expected proprioceptive feedback from an intact limb when moving to each target posture. Therefore, the participant was likely still learning to associate the tactile feedback with visual feedback of virtual hand position. Prior studies of other forms of somatosensory feedback during prosthesis use, such as non-invasive stimulation and targeted reinnervation, showed that task performance was slower with sensation than without, suggesting that participants engage more with the sensations for decision making or when focusing on the task [39, 74]. Despite evidence of improved functional accuracy with somatosensory feedback, limited studies have mentioned an increase in functional speed with touch feedback [75, 76], indicating that sensory feedback alone may not inherently increase task completion speed. Although S01’s time-to-target in the virtual environment increased with stimulation, the median time-to-target values for each condition differed by less than 1 s/target, and the participant did not report differences in difficulty or frustration between the two conditions. In addition, elicited sensations demonstrated no effect on qualitative speed, as evaluated by the occupational therapist, when completing AM-ULA functional tasks. Therefore, stimulation did not substantially degrade controller functionality.

S01 maintained a similar path efficiency compared to when stimulation was off and to prior literature regarding simultaneous and proportional myoelectric control [14, 16]. Interestingly, increasing stimulation intensity as the virtual hand aligned more with the target posture did not result in faster or more efficient movement compared to no stimulation. For both conditions, S01 watched a monitor that displayed a virtual hand responding to his attempts to move residual muscles. Previous work revealed that stimulated sensory feedback improves functional performance for tasks with obscured visual or auditory feedback [28, 29, 32, 35, 39]. Vision often dominates over other sensory modalities, including auditory and tactile feedback [77]. Visual feedback during the testing procedure allowed the participant to understand proximity to the target posture regardless of the stimulation condition.

Stimulation neither hindered nor helped myoelectric prosthesis functional performance while completing tasks of daily living, which is consistent with previous literature [35, 38]. Stimulated sensation was previously shown to improve functional performance in tasks in which vision and hearing were impaired or blocked [28, 29, 32, 35, 39]. The AM-ULA test may not adequately capture potential benefits from stimulated sensation, such as reduced reliance in visual feedback. Previous literature has shown that prosthesis users increase the duration of gaze fixation on the prosthetic hand compared to able-bodied individuals [23]. As the AM-ULA does not evaluate reliance on visual feedback or gaze patterns, the effect of iSens®-provided sensations on gaze patterns during functional tasks remains unknown and a potential area for further research. Additionally, the human hand can move about 20 DOF [78], greater than the 3 DOF controller demonstrated here, and contains a high density of mechanoreceptors to convey rich touch information [79], increased compared to the number of force sensors on the prosthesis. Increasing the number of myoelectric prosthesis DOFs and density of prosthesis force sensors will be integral for improving the congruency of expectations and reality of prosthesis movements and touch feedback.

Although stimulated sensory feedback did not improve AM-ULA performance scores, S01 rated the presence of stimulated sensation as helpful for completing AM-ULA tasks. This result aligns with previously outlined positive views regarding the helpfulness of sensory feedback [37]. In contrast to the participant’s views regarding the helpfulness of sensation, survey scores for perceived performance, confidence, difficulty, and frustration did not change with the addition of sensation. While one explanation for this discrepancy is that the participant chose to identify sensation as helpful due to confirmation bias for the specific experiment, S01 also had bias towards favoring sensation due to his extensive experience with sensory restoration for more than 10 years, which shaped his overall view of sensation benefits. The overall positive views associated with upper limb prosthesis users experiencing sensory feedback further supports the need to integrate somatosensory feedback into commercial prostheses to fulfill unmet user needs. Another caveat of the survey response results involves the lack of validation of the wording for each question, which would require administration to a larger patient population. The survey questions likely did not fully capture S01’s experiences when completing AM-ULA tasks, as previous work demonstrated increased perceived ability and confidence with the addition of sensation [35, 36].

Thus far, participants have only used the iSens® system in a laboratory setting. Continuous use of myoelectric prostheses at home allows prosthesis users to learn the controller and improve functional performance across time [80]. Using a sensory-enabled prosthesis at home has been shown to increase perceived naturalness of sensation, quality of life, embodiment, as well as other psychosocial factors, but thus far, these studies have only been conducted with percutaneous or non-invasive systems [36–39, 81]. A home trial of the fully implanted iSens® system is needed to fully understand the benefits of iSens® compared to clinically prescribed myoelectric prostheses. Given the favorable results of this initial performance evaluation, our team is starting a home trial in which one participant uses an advanced prosthesis with iSens® for daily life.

### The iSens® system addresses a clinical gap in upper limb loss rehabilitation

To the authors’ knowledge, the iSens® system provides the first non-percutaneous solution for bidirectional upper limb prostheses. The most similar prior system is the clinically tested Osseointegrated Human–Machine Gateway (e-OPRA), which provides sensory stimulation of peripheral nerves and EMG recording for persons with limb loss. The e-OPRA system uses an osseointegration interface as a wired connection for 16 conductors to send information between the implanted components and an external neural interface processor [81]. Several fully implanted technologies have focused on myoelectric recording for limb loss, such as the Myoplant [82], Myoelectric Implantable Recording Array (MIRA) [83], and Implantable Myoelectric Sensors (IMES®) [84]. The Myoplant and MIRA have been tested in animal models whereas IMES has been tested clinically. However, these devices do not provide sensory feedback to the user. Intended for individuals with paralysis, BIONic Neurons (BION®) stimulates to activate muscles [85], and the Implanted Stimulator Telemeter (IST-12) [86] and Networked Neuroprosthesis (NNP) [87] use a bidirectional approach by recording myoelectric signals and stimulating to activate muscles, of which all three devices have been tested in humans. However, these systems are primarily intended to restore muscle function to people with paralysis and have not been studied in the context of restoring sensory feedback. While several fully implantable central nervous system stimulation and recording devices exist for treating conditions such as Parkinson’s disease and paralysis, these devices are not intended for limb loss. Thus, despite the existence of various fully implanted rehabilitation technologies, none offer bidirectional sensory feedback and myoelectric control for limb loss with sufficient channel count. iSens® fills a clinical gap to provide the psychosocial and functional benefits of implanted sensorimotor interfaces for limb loss while avoiding the challenges of percutaneous interfaces.

## Conclusions

In this manuscript, we present a first-in-human implementation of iSens®, a bidirectional, wirelessly connected device for restoring somatosensation and myoelectric control for upper limb loss. This work provides an important first step in translation of neural interfacing sensorimotor restoration technologies for the field of prostheses, which can be applied to both upper and lower extremity bidirectional prostheses.

## Supplementary Information

Additional file 1: Supplementary tables and figures.

## Data Availability

The datasets supporting the conclusions of this article are available from the corresponding author upon request.

## Abbreviations

iSens®: Implanted Somatosensory Electrical Neurostimulation and Sensing
FINE: Flat interface nerve electrode
C-FINE: Composite flat interface nerve electrode
TIM: Tetra intramuscular
EMG: Electromyogram
DOF: Degree-of-freedom
BLE: Bluetooth low energy
RSSI: Received signal strength indicator
INC: Implanted neural controller
DARPA: Defense Advanced Research Projects Agency
HAPTIX: Hand proprioception and touch interfaces
VA: Veterans affairs
WFL: Waveform length
MAV: Mean absolute value
ANN: Artificial neural network
AM-ULA: Activities measure for upper limb amputees
MDC: Minimum detectable change
e-OPRA: Osseointegrated human–machine gateway
MIRA: Myoelectric implantable recording array
IMES®: Implantable myoelectric sensors
BION®: BIONic neurons
IST-12: Implanted stimulator telemeter
NNP: Networked neuroprosthesis
S01: A study participant
S02: A study participant
EDC: Extensor digitorum communis muscle
ECRL: Extensor carpi radialis longus muscle
ECRB: Extensor carpi radialis brevis muscle
ECU: Extensor carpi ulnaris muscle
FPL: Flexor pollicis longus muscle
FCR: Flexor carpi radialis muscle
FCU: Flexor carpi ulnaris muscle
FDS: Flexor digitorum superficialis muscle
EIP: Extensor indicis proprius muscle
APL: Abductor pollicus longus muscle
FDP: Flexor digitorum profundus muscle
