# Supplementary material for: First-in-human implementation of a bidirectional somatosensory neuroprosthetic system with wireless communication
Source: J Neuroeng Rehabil. 2025 Apr 23;22:90. doi: 10.1186/s12984-025-01613-z (PMC12020317; doi:10.1186/s12984-025-01613-z)
Supplement: Supplementary file 1 — Additional file 1: Supplementary tables and figures. [file 12984_2025_1613_MOESM1_ESM.pdf]

Additional File 1: Supplementary materials

Supplementary Tables

Table S1: Implanted electrode anatomical locations.

	S01		S02	
	Anatomy	Device dimensions	Anatomy	Device dimensions
Muscles (TIM bipolar channel count)	extensor digitorum communis m. (4)	25 cm	extensor carpi radialis longus m. (1)<br>supinator m. (1)<br>extensor digitorum communis m. (2)	25 cm
	supinator m. (1)<br>extensor carpi radialis longus m. (1)<br>extensor carpi radialis brevis m. (1)<br>extensor carpi ulnaris m. (1)	25 cm	extensor digitorum communis m. (1)<br>extensor indicis proprius m. (1)<br>abductor pollicus longus m. (1)<br>extensor carpi ulnaris m. (1)	35 cm
	flexor pollicis longus m. (1)<br>pronator teres m. (1)<br>flexor carpi radialis m. (1)<br>flexor carpi ulnaris m. (1)	15 cm	flexor carpi ulnaris m. (1)<br>flexor digitorum superficialis m. (3)	25 cm
	flexor digitorum superficialis m. (4)	15 cm	pronator teres m. (1)<br>flexor pollicis longus m. (1)<br>flexor carpi radialis m. (1)<br>flexor digitorum profundus m. (1)	25 cm
Nerves (C-FINE count)	median n. (2)	10x1.5 mm	median n. (2)*	10x1.5 mm
	ulnar n. (1)	10x1.0 mm	ulnar n. (1)	10x1.0 mm
	radial n. (1)	10x1.0 mm	radial n. (1)*	10x1.5 mm

Muscles and nerves implanted with TIMs and 16 C-FINEs for each study participant, respectively. *Note that one of S02's Smart Stim Leads, connected to two 16 C-FINEs on the median and radial nerves, was not able to successfully communicate with the INC after the original implant procedure.

Table S2: Peripheral nerve stimulation pulse parameters implemented while completing the AM-ULA.

Sensor location	Percept location	PA (Trial 1)	PA (Trials 2-3)	PW	PF (Trial 1)	PF (Trials 2-3)
Thumb	Thumb	0.33 mA	0.30 mA	250 ms	45, 70, 100 Hz	75, 100, 150 Hz
Middle	Index	0.37 mA	0.30 mA	250 ms	30, 70, 100 Hz	30, 70, 100 Hz
Palm	Palm, Dorsal hand	0.27 mA	0.26 mA	250 ms	30, 70, 100 Hz	50, 75, 100 Hz

PW was constant during each experimental session. PF increased to each specified value based on pressure applied to each DEKA Luke arm prosthesis sensor. Sensor thresholds were evenly spaced based on calibration prior to testing. When the sensor value exceeded a threshold, the PF increased to the next highest value. PA was adjusted prior to the start of each experimental session to achieve sensation intensities within a comfortable and perceivable range, and PA was held constant once the test began.

Table S3: Survey questionnaire administered during the AM-ULA.

Survey Question	Minimum Score	Maximum Score
1. How well were you able to perform this task?	0 = performed very poorly	4 = performed as well as I could prior to my amputation
2. How confident were you performing this task?	0 = not at all	4 = extremely confident
3. How difficult was performing this task?	0 = very easy	4 = very difficult
4. How frustrated were you regarding your prosthetic control during the task?	0 = not at all	4 = extremely frustrated
5. *If stimulation was active:* To what extent did the sensation help you perform the task?	0 = not at all	4 = a great deal

A researcher stated questions after each AM-ULA task, S01 verbally reported his answers, and the researcher recorded responses. Each item in the survey was rated on a 5-point scale.

Table S4: Categorized quality descriptors.

Subject	Tactile		Proprioception		Pain	
S01	Vibration	67%	Tensing	12%	Aching	3%
	Pressure	31%	Squeezing	9%	Pain	2%
	Fluttering	14%	Contraction	9%	Sharp	1%
	Tickling	4%	Tight	3%	Uncomfortable	0.8%
	Tingle	2%	Pulling	2%	Burning	0.8%
	Dull	0.8%	Moving	0.8%	Hurting	0.4%
	Tapping	0.8%			Stinging	0.4%
	Pushing	0.8%			Pins and needles	0.4%
	Electrical	0.4%				
	Touch	0.4%				
S02	Vibration	66%	Contraction	18%	Sharp	2%
	Electrical	45%	Tight	3%	Shocking	0.8%
	Buzzing	18%	Moving	2%	Pins and needles	0.8%
	Tingle	13%	Tensing	0.8%		
	Dull	4%	Squeezing	0.8%		
	Gentle	3%				
	Pulsing	2%				
	Smooth	2%				
	Pressure	0.8%				
	Touch	0.8%				
	Traveling	0.8%				

List of all quality descriptors participants reported categorized into tactile, proprioceptive, and pain categories, and percent of trials in which each descriptor was reported across all sessions and all C-FINE contacts. Participants reported descriptors during each experimental session from C-FINE-evoked sensations at threshold perception. Participants freely reported sensation qualities that described their perceived sensation, without a word bank.

Supplementary Figures

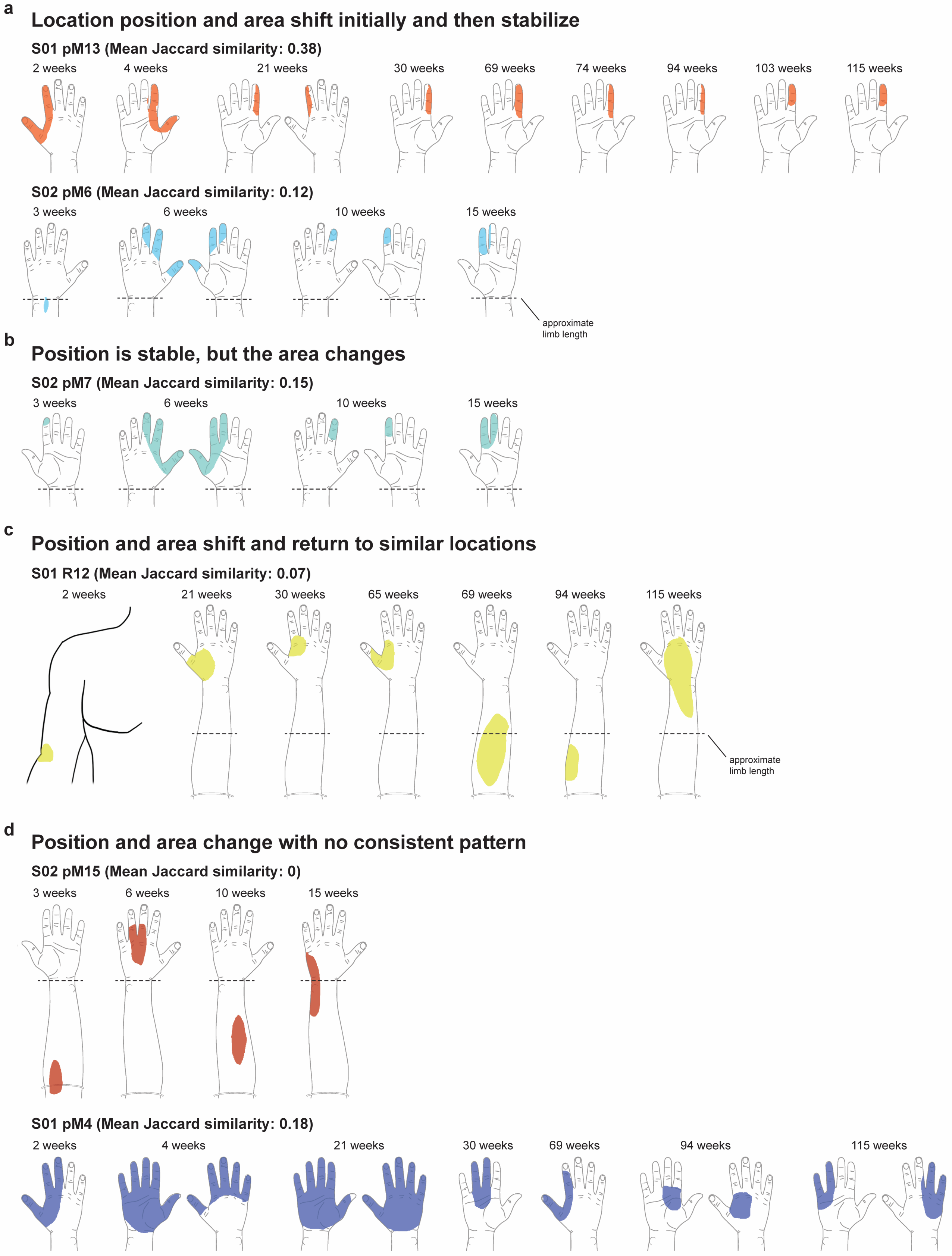

Fig. S1: Examples of percept locations across time. a) S01's pM13 contact elicited sensation originally on the back of the thumb and index finger, but the location shifted to the palmar side of the thumb and remained stable up to 115 weeks post-implant. S02 originally experienced sensation in the residual limb wrist through pM6, but the sensation shifted to the index finger during subsequent sessions. The area of sensation varied during the last three sessions, but percepts continued to be centered at the index finger. b) S02's pM7 contact evoked sensation at the index finger during all sessions, but percept area sizes varied, and reports of adjacent percept areas for the middle finger and thumb changed across time. c) Sensations evoked through S01's R12 contact shifted between the residual limb and dorsal hand across time, but shifting often returned to similar locations as reported previously. d) Both S01 and S02 reported percept locations across time for contacts that did not show a consistent shifting pattern or stable locations, such as S02's pM15 and S01's pM4. S02 demonstrated no overlapping locations for pM15 percepts across time. S01's percept locations elicited by pM4 occasionally overlapped with previously reported percept locations, but overall, percept locations did not show consistency across time for this contact.

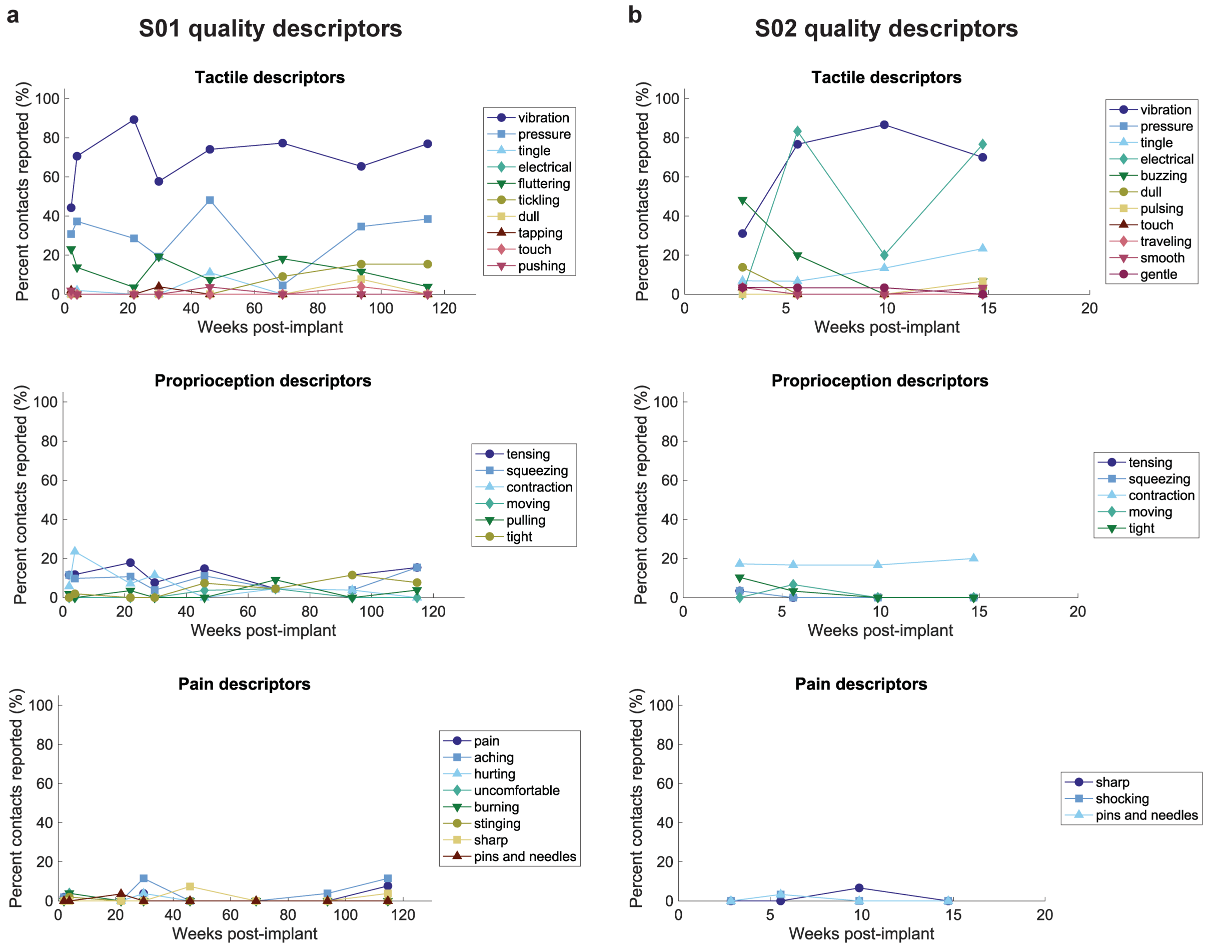

Fig. S2: Percent of contacts associated with individual quality descriptors across time. Percent of contacts in which a) S01 and b) S02 reported quality descriptors across time, categorized by tactile, proprioceptive, and pain descriptors. Participants freely chose quality descriptors, without a word bank.

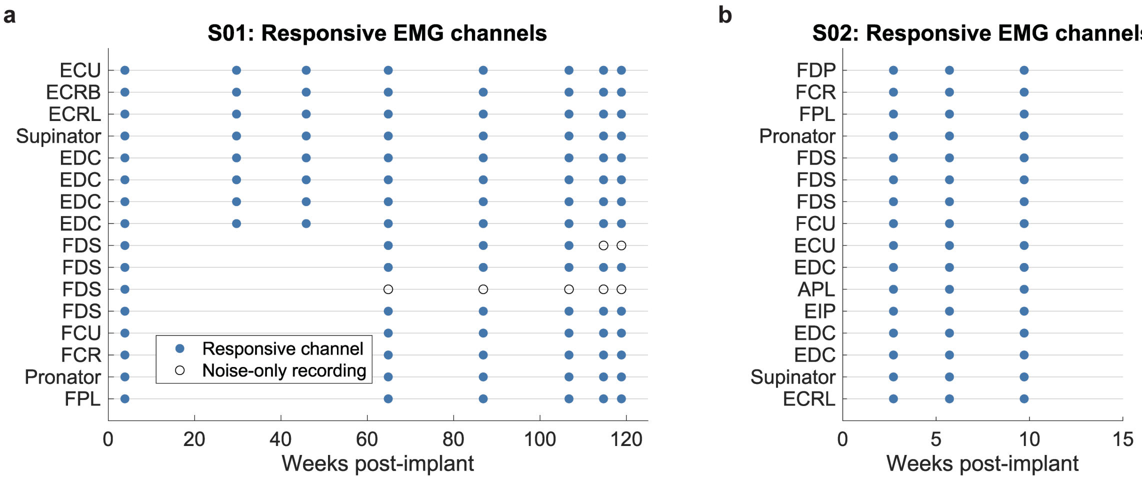

Fig. S3: Responsive EMG channels across time. Responsive EMG channels for a) S01 and b) S02. Responsive channels record increased signal amplitudes during voluntary contractions and decreased signal amplitudes during rest phases. Unresponsive channels record only noise and do not show modulation in amplitude during voluntary movements. Labels on the y-axis represent muscles where each bipolar EMG channel is implanted.

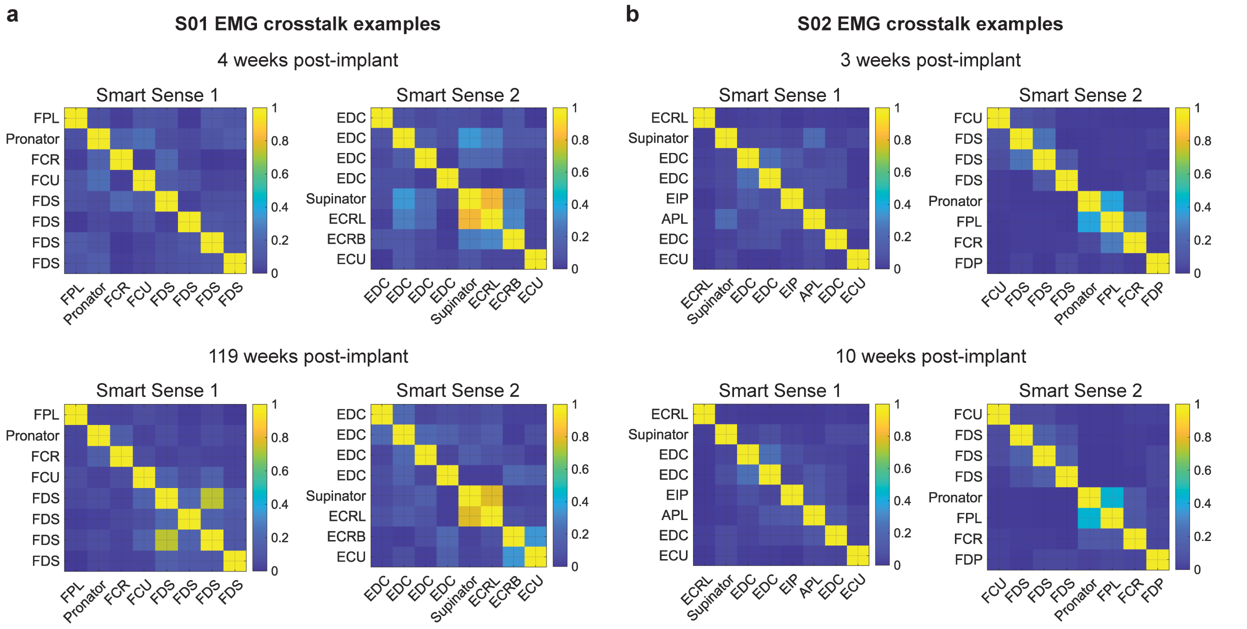

Fig. S4: Smart Sense EMG channel cross correlation confusion matrix examples. Cross correlation confusion matrices during initial (top) and later (bottom) experimental sessions for a) S01 and b) S02. Confusion matrices are reflected about the diagonal and show the amount of crosstalk between each pair of EMG channels on a given Smart Sense Lead.

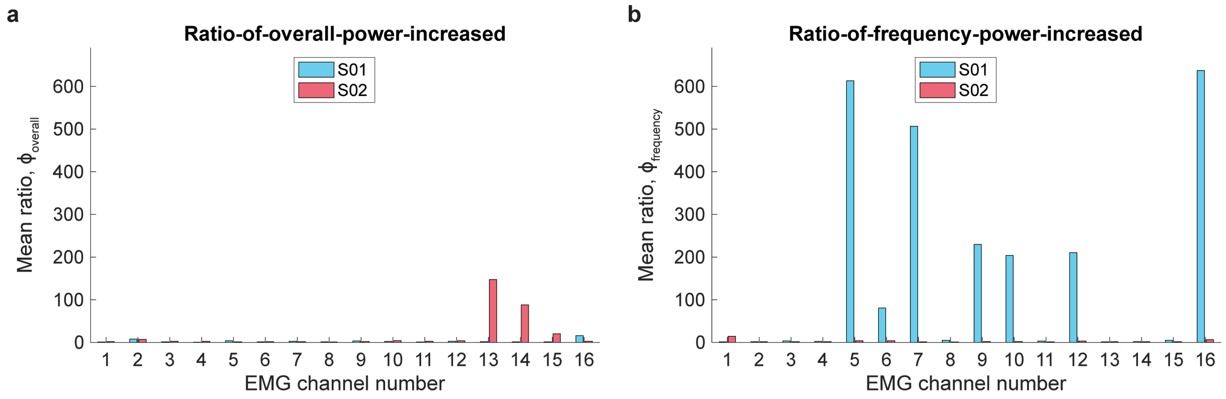

Fig. S5: Signal power increase ratios. a) Ratio-of-overall-power-increased, averaged across trials for each C-FINE contact tested, for the median power increased at all frequencies other than within ±5± Hz of the stimulation frequency (70 Hz) and harmonic frequencies at all time points. b) Ratio-of-frequency-power-increased, averaged across trials for each C-FINE contact tested, for the median power increased across the stimulation frequency (70 Hz) and harmonic frequencies at all time points. Note that S01's channel 6 was determined to be unresponsive based on low WFL variance regardless of voluntary motion.

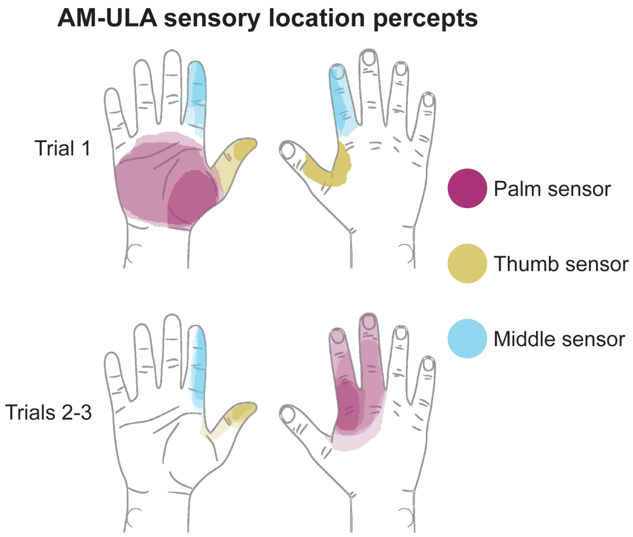

Fig. S6: Reported sensory locations prior to completing the AM-ULA. S01's location percepts experienced just prior to using the real-time, 3 DOF control algorithm and DEKA Luke arm when completing the AM-ULA. Percepts at all intensity levels are shown, between minimum perception to mid-comfortable intensities. Shaded areas are proportional to the number of times the participant circled the pixels when stimulating through each C-FINE contact.
